# Beyond Single-Reference Fixed-Node Approximation in *Ab Initio* Diffusion Monte Carlo Using Antisymmetrized Geminal Power Applied to Systems with Hundreds of Electrons

**DOI:** 10.1021/acs.jctc.4c00139

**Published:** 2024-05-24

**Authors:** Kousuke Nakano, Sandro Sorella, Dario Alfè, Andrea Zen

**Affiliations:** † Center for Basic Research on Materials, National Institute for Materials Science (NIMS), Tsukuba, Ibaraki 305-0047, Japan; ‡ International School for Advanced Studies (SISSA), Via Bonomea 265, 34136 Trieste, Italy; § Dipartimento di Fisica Ettore Pancini, Università di Napoli Federico II, Monte S. Angelo, 80126 Napoli, Italy; ∥ Department of Earth Sciences, University College London, Gower Street, London WC1E 6BT, U.K.; ⊥ Thomas Young Centre and London Centre for Nanotechnology, 17-19 Gordon Street, London WC1H 0AH, U.K.

## Abstract

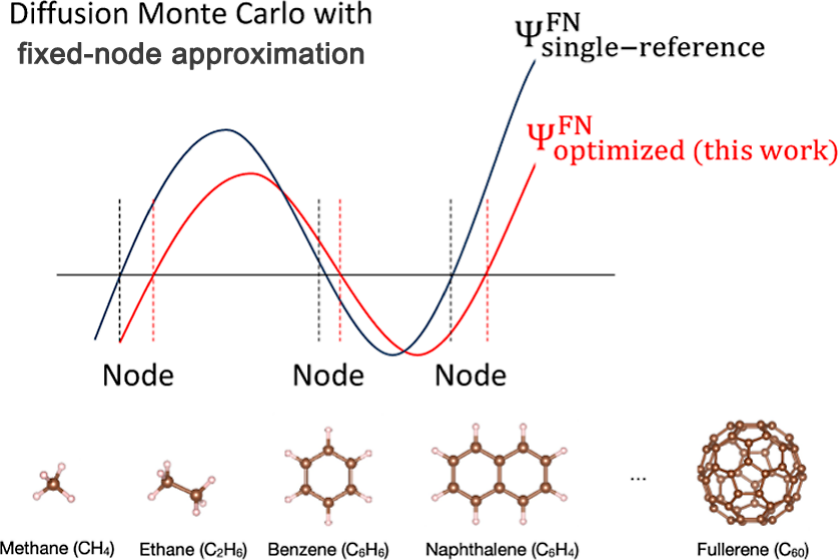

Diffusion Monte Carlo (DMC) is an exact technique to project out the ground state (GS) of a Hamiltonian. Since the GS is always bosonic, in Fermionic systems, the projection needs to be carried out while imposing antisymmetric constraints, which is a nondeterministic polynomial hard problem. In practice, therefore, the application of DMC on electronic structure problems is made by employing the fixed-node (FN) approximation, consisting of performing DMC with the constraint of having a fixed, predefined nodal surface. How do we get the nodal surface? The typical approach, applied in systems having up to hundreds or even thousands of electrons, is to obtain the nodal surface from a preliminary mean-field approach (typically, a density functional theory calculation) used to obtain a single Slater determinant. This is known as single reference. In this paper, we propose a new approach, applicable to systems as large as the C_60_ fullerene, which improves the nodes by going beyond the single reference. In practice, we employ an implicitly multireference ansatz (antisymmetrized geminal power wave function constraint with molecular orbitals), initialized on the preliminary mean-field approach, which is relaxed by optimizing a few parameters of the wave function determining the nodal surface by minimizing the FN-DMC energy. We highlight the improvements of the proposed approach over the standard single-reference method on several examples and, where feasible, the computational gain over the standard multireference ansatz, which makes the methods applicable to large systems. We also show that physical properties relying on relative energies, such as binding energies, are affordable and reliable within the proposed scheme.

## 1 Introduction

Ab initio electronic structure calculations, which compute the electronic structure of materials nonempirically, have become an essential methodology in the materials science and condensed matter physics communities. Density functional theory (DFT), a mean-field approach which was originally proposed by Kohn and Hohenberg,^1^ is the most widely used methodology for ab initio electronic structure calculations. DFT has enjoyed widespread success, despite its reliance on the so-called exchange–correlation (XC) functional, whose exact form is yet to be discovered. Although many XCs have been proposed, no functional that performs universally well for all materials is established.

Several methodologies transcend the mean-field paradigm. For example, in the quantum chemistry community, the coupled cluster method with single, double, and perturbative triple excitations,^2^ denoted as CCSD(T), is widely recognized as the gold-standard approach, balancing accuracy and computational efficiency. This technique has been employed as a reference in many benchmark tests, both for isolated and periodic systems.^2−5^ CCSD(T) is mostly applied in relatively small systems, as it becomes very computationally intensive as the simulated systems get larger (hundreds of electrons or more). Moreover, despite the many successes of CCSD(T), there are a few cases where CCSD(T) fails, mostly attributed to the multireference character of a chemical system (strong correlation) and where other methods, more expensive computationally, are needed.^6^ A different approach, adopted by the condensed matter community as the gold standard, is the diffusion Monte Carlo (DMC) method.^7^ DMC has good scaling with the system size and it uses algorithms that can be parallelized with little or no efficiency loss, fully exploiting modern supercomputers and making relatively large systems treatable.

CCSD(T) and DMC predictions typically show consensus in the computed physical properties, such as heats of formation and binding energies, and good agreement with experiments.^5,8−13^ It was believed that CCSD(T) and DMC would also agree on extended systems, but recent findings by Al-Hamdani et al.^12^ have unveiled discrepancies in binding energy calculations between these methods for large systems, such as a C_60_ buckyball inside a [6]-cycloparaphenyleneacetylene ring (C60@[6]CPPA). It is unclear which approach is to be trusted in these tricky cases. These findings raise a pivotal question: what is the reference approach for noncovalent interactions between large systems? To answer this question, Al-Hamdani et al.^12^ discussed possible discrepancy sources coming from uncontrollable errors existing in both CCSD(T) and DMC. Both approaches employ some approximations and have their weaknesses, and the debate is still open. To draw a more conclusive determination, one should develop a scheme which mitigates the impact of uncontrollable errors in the methods. In this work, we focus on improvements in the DMC approach that alleviate its largest source of error: the fixed-node (FN) approximation.

DMC yields the exact ground state (GS) in bosonic systems. In Fermionic systems (for instance, in electronic structure calculations), DMC suffers from the so-called negative sign problem, arising from the fact that the Fermionic GS has positive and negative regions. The negative sign problem in the DMC method for Fermions has been proven to be a nondeterministic polynomial hard problem;^14^ thus, it seems unrealistic to find a general solution at present. This problem is avoided, in practice, by modifying the DMC projection algorithm with the introduction of the FN approximation, where the projected wave function Φ_FN_ is constrained to have the nodes of a predetermined guiding function Ψ_T_. The FN approximation keeps the projected wave function Φ_FN_ antisymmetric, but Φ_FN_ is the exact GS Φ_0_ only if its nodes are exact. A general property of Φ_FN_ is that it is always the closest function to Φ_0_ within the FN constraint. For trial functions obtained from mean-field approaches, such as Hartree–Fock (HF) or DFT, it is generally believed that the error associated with the FN approximation is small and benefits from a large error cancellation in the evaluation of binding energies.^8^ However, the FN error is typically not accessible, as Φ_0_ is unknown, and this yields an uncontrollable error in FN-DMC.

In standard FN-DMC simulations, the nodal surface is given by an approximate wave function, which is typically obtained starting from a mean-field approach, such as HF or DFT. The variational principle can still be applied to FN-DMC,^a^ and so to go beyond the mean-field solution, one should optimize the given nodal surface by minimizing the FN-DMC energy *E*_FN_ (which is the expectation value of Φ_FN_), going in the direction of the exact wave function Φ_0_ and the exact energy *E*_0_. This procedure is seldomly followed in DMC simulations, especially on large systems (say, with hundreds or thousands of electrons), as it is hardly affordable computationally and the uncertainty on the optimization of the FN surface could be easily comparable, if not larger, than the binding energy under consideration. Thus, the standard approach is to just keep the nodal surface of the Slater determinant (SD) built with the Kohn–Sham orbitals obtained from a DFT calculation. While the FN surface from DFT might be suboptimal, this approach typically yields quite reliable results, especially in the evaluations of noncovalent interactions, due to very favorable error cancellations.^5,8^

In smaller systems (with say, tens of electrons), it is possible to improve the nodal surface, and the most standard approach is to use an ansatz that has more degrees of freedom than the initial SD, such as the antisymmetrized geminal power (AGP),^19−21^ the Pfaffian,^22−24^ the complete active space,^25,26^ the valence bond,^27,28^ the backflow,^23,29,30^ and multideterminant expansions,^31−38^ including methods employing neural networks and machine learning techniques.^39−45^ The standard approach here is to optimize the wave function parameters at the level of theory of variational Monte Carlo (VMC).^19,46−50^ Indeed, optimization at the FN-DMC (FN-opt) level implies further difficulties, as we will discuss below. However, optimization at the VMC (VMC-opt) level has some flaws. In VMC-opt, the object that is optimized is the variational wave function Ψ_T_, which is obtained from the product of one of the ansatzes discussed above and the Jastrow factor.^b^ The closer Ψ_T_ gets to the GS Φ_0_, the smaller its VMC energy (variational principle) and its VMC variance (zero-variance property) are. VMC-opt explores the parameters’ variational space, seeking the set which minimizes the VMC energy or the VMC variance, and it is often done by employing the VMC gradient. It is not guaranteed that the parameters obtained from VMC-opt are those giving the best possible nodal surface allowed by the employed ansatz (unless we are in the limit case where Ψ_T_ yields VMC with zero variance, such that we know that Ψ_T_ is an eigenstate of the Hamiltonian). Although this approach, in practice, gives a better nodal surface than the DFT one, it sometimes gives unreasonable outcomes, e.g., it overestimates binding energies, as revealed in this work. It would be desirable, instead, to implement an optimization at the FN-DMC level of theory, where the parameters of the function Ψ_T_ giving the nodal surface are optimized so as to minimize the FN energy. This would guarantee to find the best nodal surface allowed by the adopted wave function ansatz. To the best of our knowledge, the first attempt to directly optimize the variational parameters included in a trial wave function at the FN-DMC level was done by Reboredo et al.^51^ in the ab initio framework. They proposed a way to iteratively generate new trial wave functions to get a better nodal surface. They generalized the method to excited states^52^ and finite temperatures^53^ and also applied for large systems such as C_20_.^54^ Very recently, McFarland and Manousakis^55^ reported successful energy minimizations with approximated and exact FN gradients. They proposed to optimize nodes using a combination of FN gradients and the projected gradient descent (PGD) method. The PGD method works for Be, Li_2_, and Ne using all-electron DMC calculations,^55^ while it has been successful only for small molecules.

When it comes to optimizing the nodal surface of a large system, the main problem is that the number of variational parameters determining the nodal surface often scales more than linearly with the size of the system. For instance, the number of variational parameters in the determinant part of the AGP ansatz scales with *O*(*L*^2^), where *L* is the number of basis functions in a system. It makes the parameter space to be optimized so complex that the optimization is easily trapped in local minima and one cannot find the true GS. Moreover, since the optimization algorithms are stochastic, there is always an additional uncertainty on the optimized parameters, which are not going to be exact and the corresponding DMC energy has therefore an optimization bias. The optimization bias increases with the system size and with the number of variational parameters and can be reduced only at the cost of increasing the statistical sampling (and the computational cost). The evaluation of binding energy implies the difference between two or more DMC energies, and it is often a tiny fraction of the total energy. Therefore, the optimization uncertainty can often be comparable to the binding energy, making the evaluation of the interaction energy unreliable. Moreover, we need to verify that the adopted approach satisfies basic physical properties, such as being size-consistent.^c^ At the VMC level, the size consistency is a property of the wave function ansatz employed, and it depends on the optimization procedure. At the FN-DMC level, size consistency might depend on some choices on the algorithm,^56^ on the ansatz of the wave function providing the FN constraint, and on the optimization.

In this paper, we propose a scheme which aims to address these issues. In particular, our scheme satisfies the following points: (i) it is systematically more accurate than the standard approach of employing a single SD, (ii) it is size-consistent, and (iii) it is applicable also to large systems. The idea underlying the present work is the combination of the AGP wave function consisting of molecular orbitals (MOs), dubbed AGPn,^21^ the use of natural orbitals (NOs), and the optimization of its nodal surface using FN gradients on a selected subset of the AGPn parameters. In particular, we initialize the orbitals in the AGPn wave function using NOs, which are kept fixed afterward, such that only the coefficients combining them are optimized to relax the nodal surface. We call this scheme the fixed node antisymmetrized geminal power active space (FNAGPAS). Since the orbitals are fixed, this results in a much smaller number of variational parameters in the ansatz; thus, one can apply it for larger systems, such as C_60_ fullerene. We show that our scheme gives a better nodal surface (i.e., a lower energy in the FN-DMC calculation) compared to the typical Slater–Jastrow ansatz, and it reliably describes also strongly correlated systems (such as diradicals). We show that the use of FN-opt is important to fulfill the size-consistency property.

## 2 FNAGPAS Scheme

**Figure 1:**
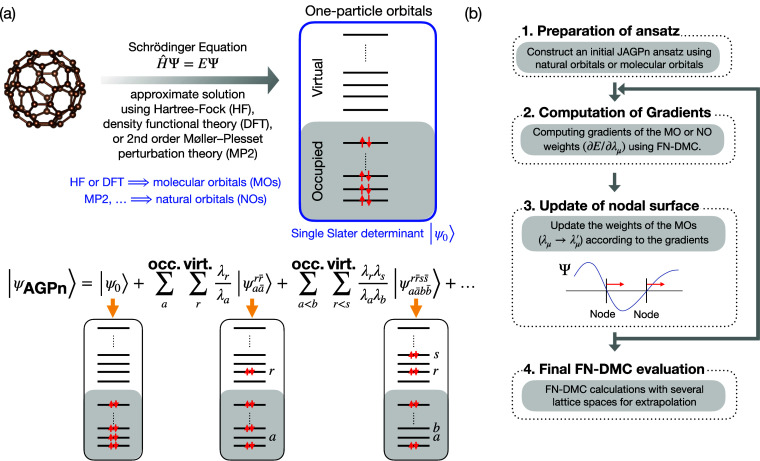
Panel a: Schematic illustration of the FNAGPAS scheme. We perform a preliminary mean-field calculation to obtain MOs, followed by a correlated calculation yielding NOs. The AGPn ansatz corresponds to a multideterminant expansion built on the NOs and depending on the coefficients λ_*i*_ associated with each orbital *i* and optimized in order to minimize the FN energy. Panel b: Flowchart illustrating the FNAGPAS scheme workflow.

We describe here the scheme that we suggest to improve the accuracy of FN-DMC over the traditional single-determinant Slater–Jastrow ansatz. The key idea is the combination of the AGPn,^21^ which is the AGP wavefunction constraint with MOs, and the optimization of the ansatz using approximated FN gradients.^55^ We describe the ansatz in the following section, assuming an unpolarized system for simplicity. The schematic illustration explaining the key concept and its workflow is shown in Figure 1.

The real-space quantum Monte Carlo (QMC) typically employs a many-body wave function ansatz Ψ written as the product of two terms, Ψ_QMC_ = Φ_AS_ × exp *J*. The term exp *J*, conventionally dubbed Jastrow factor, is symmetric, and the term Φ_AS_ is antisymmetric. The Jastrow factor is explicitly dependent on electron–electron distance and often includes electron–nucleus and electron–electron–nucleus terms.^d^ The nodal surface of a wave function is determined by the antisymmetric part Φ_AS_ (because exp *J* ≥ 0). Thus, in FN-DMC, the accuracy of the results depends crucially on the quality of the nodes of Φ_AS_.

The antisymmetric part of a trial wave function is initially constructed from a mean-field self-consistent-field (SCF) approach, such as DFT or HF. The standard QMC setup in large systems is to define Φ_AS_ as the single SD obtained from such preliminary SCF calculations. The corresponding Ψ_QMC_ is dubbed JSD. Therefore, the nodes of JSD are predefined before any QMC calculation and unrelaxed. Initializing the SD using different setups for the SCF calculations (e.g., different exchange–correlation functionals) leads to slightly different total energies, but most of the times, the interaction energies (which are evaluated from energy differences between two or more systems) are almost unaffected by the details of the preliminary SCF calculation, especially for weak noncovalent interactions. This is an indication that there is an almost perfect cancellation of the error induced by the FN approximation within the JSD ansatz, provided that the SD is initialized consistently in all systems.

However, changing the setup of the SCF calculation only allows the nodes to move within the variational freedom of a single SD. By contrast, giving Φ_AS_ the variational freedom to relax the nodes beyond the JSD ansatz leads to an improvement of the FN-DMC total energy of the system,^58^ and possibly, also the interaction energies could change. The challenge that we take here is to generalize the ansatz in a way that large systems are still doable.

Here, we suggest to use the AGP ansatz as Φ_AS_. AGP is an implicitly multideterminant ansatz,^56,59^ which corresponds to a constrained zero-seniority expansion, as illustrated schematically in Figure 1. The evaluation of an AGP function can be reduced to the computation of a determinant; therefore, the AGP ansatz is computationally comparable to an SD (differently from explicitly multideterminant functions), thus ensuring the cubic scaling with the system size of both the variational and FN algorithms.^e^ The AGP ansatz for a system of *N*_el_ electrons is
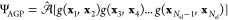
1(we are assuming for simplicity an unpolarized system with an even number of electrons, but the ansatz can be generalized as discussed in ref (19)), where  is the antisymmetrization operator and the function *g* is the geminal function , which is a pairing function between two electrons with coordinates **x**_1_ and **x**_2_ forming a spin singlet. The spatial part *f*(**r**_1_, **r**_2_) is symmetric, and it can be written in terms of a basis set {χ_μ_} for the single-electron orbital space as follows:
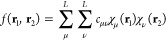
2where μ and ν run over all the *L* basis orbitals and *c*_μν_ are variational parameters. Notice that in general, *L* ≫ *N*, and the number of variational parameters *c*_μν_ is equal to *L*^2^. The parameters define a *L* × *L* symmetric matrix **C** (the symmetry of *f* implies *c*_μν_ = *c*_νμ_), so there is an orthogonal transformation **U** which diagonalizes **C** and allows rewriting *f* as
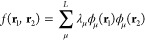
3where ϕ_μ_ = *∑*_ν_*U*_μν_χ_ν_. With no loss of generality, we can assume that λs are ranked in a decreasing order of their absolute value. Notice that if only the first *N*_el_/2 λs are different from zero, then Ψ_AGP_ corresponds to a single SD built on the orbitals  occupied with both spin-up and spin-down electrons. Since such an SD built on orbitals from an SCF calculation is the standard QMC setup, and it typically delivers good results, we tried to relax the nodes by considering a subset *n*_orb_ (larger than *N*_el_/2 but ≪ *L*) of the orbitals obtained from the SCF calculation. This is what we call the AGPn ansatz.

For efficient and effective use in QMC, the AGP and AGPn functions shall be multiplied by a Jastrow factor, yielding the so-called JAGP and JAGPn functions. The Jastrow factor can have the same variational form used also in JSD, which allows for the JSD, JAGP, and JAGPn functions to satisfy the cusp conditions and to effectively recover the dynamical correlations. Indeed, the main improvement of JAGP and JAGPn over JSD is their ability to capture static correlations, yielding to qualitatively different results on systems with an underlying multireference character, both at the variational and at the FN level of theory.^56,59^ The optimization of the parameters in the Jastrow is usually quite a feasible problem also on large systems, as their number does not grow uncontrollably with the size of the system. In practice, every QMC code implements a slightly different functional form of the Jastrow, but they share the general features mentioned above. The QMC code used in this work is TurboRVB,^57^ an open-source package. The Jastrow factor implemented in TurboRVB (described in ref (57)) has a number of parameters growing linearly with the size of the system (as shown in the Results and Discussion section).

In this work, we keep the orbital frozen and optimize the coefficients λ_1_, ..., λ_*n*_orb__ of the JAGPn ansatz using FN-DMC gradients. A similar idea, but at the variational level, was also mentioned in a seminal work by Casula and Sorella to describe the BCS pairing function in iron-based superconductors.^60^ JAGPn dramatically reduces the number of variational parameters with respect to the JAGP ansatz, such that the optimization of the JAGPn function is doable even in pretty large systems, in contrast to JAGP which is affordable only on relatively small systems. Nevertheless, employing JAGPn significantly improves the FN-DMC energy (as well as the variational QMC energy) over the results within the traditional JSD function, as we will show in the results section. Of course, the JAGP ansatz has higher variational freedom than JAGPn, so JAGP can in principle improve further over JAGPn. However, in practice, we observe that FN-DMC energies obtained from the JAGP ansatz are comparable to those obtained from JAGPn on small systems (and both JAGP and JAGPn are significantly better that JSD), while, in large systems, JAGP is unaffordable because the optimization can be stuck at local minima at the variational level and can become unstable at the FN level. The latter instability is probably due to insufficient signal-to-noise ratios^61^ that the QMC optimization always suffers from, but the origin of the instability is yet unclear. On intermediate systems, we notice that JAGP FN-DMC energy is worse than the JAGPn FN-DMC energy, as a clear indication that despite the higher variational freedom on JAGP, the optimization of that many parameters is not converging and there is too much noise on the parameters.

The main problem of the AGP ansatz (and AGPn) is that it is not size-consistent at the variational level of theory, but JAGP (JAGPn) is size-consistent if we employ a very flexible Jastrow factor.^62,63^ Since the FN-DMC corresponds to applying an infinitely flexible Jastrow factor to the determinant part, optimizing the AGPn parameters at the FN level ensures the size consistency of our approach.

A crucial point to make JAGPn almost as accurate as JAGP, despite employing only a small number *n*_orb_ of parameters λs, is to carefully choose the orbitals. We notice that the virtual orbitals obtained from SCF calculations are typically not optimal, as we need a large number of them (of the order of *L*) to converge to the best JAGPn FN energy. Moreover, if we cannot afford a systematic test of the convergence of *n*_orb_ for each system of interest, it is difficult to define a sensible criterion to decide which *n*_orb_ to pick. We solved both the problems by employing NOs for expanding the pairing function, instead of using MOs. NOs were constructed from second-order Møller–Plesset (MP2) calculations. This is because the MP2 unoccupied orbitals incorporate perturbation effects and are physically better than those obtained with HF or DFT,^64^ as shown in the Supporting Information. More specifically, we constructed NOs by diagonalizing the density matrix obtained by MP2 calculations. We also notice that a method to construct NOs should be affordable also for large systems. This is also a reason why we chose MP2 for constructing NOs in this study. In practice, from the weight of the NOs, we can easily define a cutoff value to select *n*_orb_ on each system, and we notice that we get to converged results already with a value of *n* that is not much larger than *N*_el_/2 (*n*_orb_ = *N*_el_/2 would correspond to a single SD).

## 3 Computational Details

We applied our scheme to planar and twisted ethylenes, eight hydrocarbons (CH_4_, C_2_H_4_, C_2_H_6_, C_6_H_6_, C_10_H_8_, C_14_H_10_, C_18_H_12_, and C_20_H_10_), the C_60_ fullerene, and water–methane dimer (see Supporting Information for their coordinates). The number of valence electrons treated in this study is 12, 12, 8, 12, 14, 30, 48, 66, 84, 90, 240, and 16, respectively. The MP2 calculations (HF and DFT calculations for comparison) to generate nodal surfaces of trial wave functions were performed using PySCF v.2.0.1.^65,66^ The trial wave functions were converted to the TurboRVB wave function format using TurboGenius^67^ via TREXIO(68) files. We employed the cc-pVQZ basis set accompanied by the ccECP pseudopotentials^69^ for the eight hydrocarbons and C_60_ fullerene, while the cc-pVTZ basis set accompanied by the ccECP pseudopotentials^69^ for the water–methane and for the torsion calculation of ethylene. We employed [3s], [3s1p], and [3s1p] primitive Jastrow basis for H, C, and O atoms, respectively. The Jastrow factor and the weights of the NOs in the pairing function (i.e., the nodal surface of a wave function) were optimized using the stochastic reconfiguration method^70^ implemented in TurboRVB^57^ with an adaptive hyperparameter.^71^ The Jastrow factor was optimized only with VMC gradients, and it was held fixed during optimization with FN gradients. The FN gradients were computed from a standard walker distribution using mixed estimators, which corresponds to method A in ref (55). The lattice-discretized version of the FN-DMC calculations (LRDMC)^72,73^ was used in this study. The single-shot LRDMC calculations were performed by the single-grid scheme^72^ with lattice spaces *a* = 0.30, 0.25, 0.20, and 0.10 Bohr, and the energies were extrapolated to *a* → 0 using *f*(*a*^2^) = *k*_4_·*a*^4^ + *k*_2_·*a*^2^ + *k*_0_. The LRDMC calculations for computing those gradients were performed by the single-grid scheme^72^ with lattice space *a* = 0.20 Bohr. The determinant locality approximation (DLA)^18^ was employed for the LRDMC calculations.^f^ We notice that the LRDMC framework guarantees the variational principle even with the presence of nonlocal pseudopotentials, as proven in the Appendix. The molecular structures are depicted using VESTA.^74^

## 4 Results and Discussion

### 4.1 FNAGPAS Captures Strong Correlation

**Figure 2:**
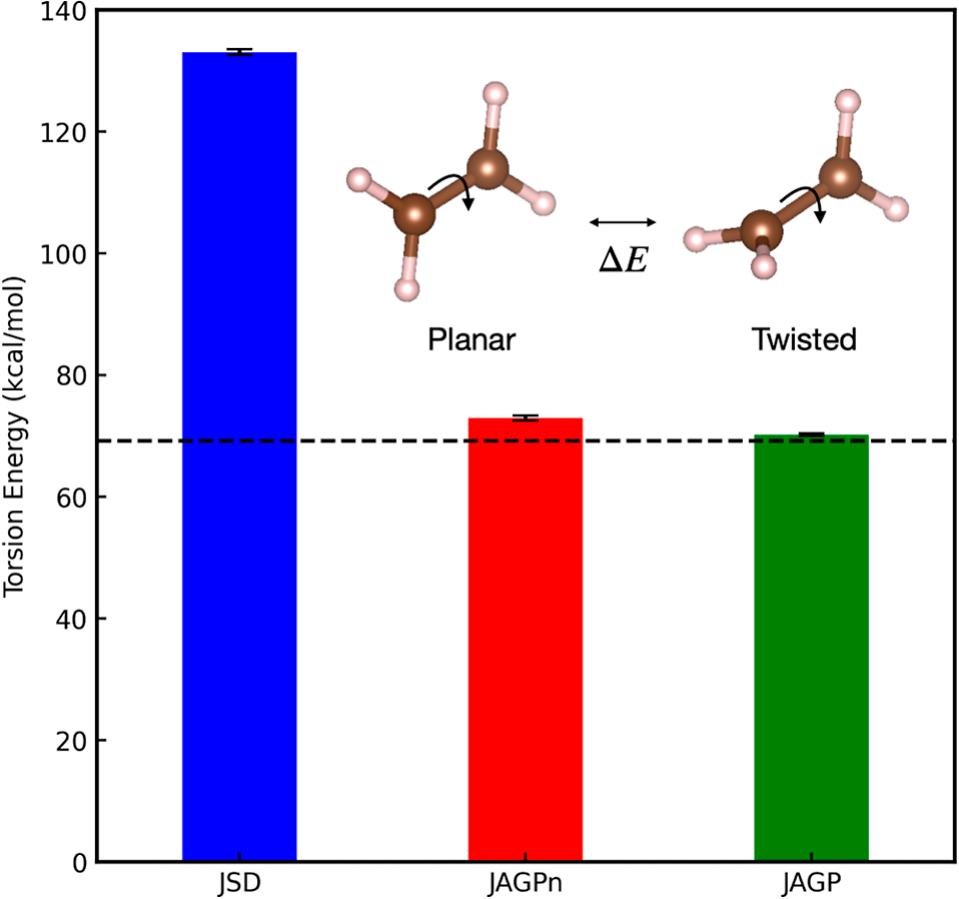
Torsion energy of ethylene from FN-DMC with JSD, JAGPn or JAGP wave functions. The values of JAGP and MR-CISD + Q (horizontal broken line) are taken from refs (59 and 76), respectively. The inset shows the structure of the planar and twisted ethylene.

We show that the proposed FNAGPAS is able to incorporate the correlation effect that the JSD ansatz cannot do at all. We apply our scheme for the torsion energy estimation of ethylene (C_2_H_4_). The torsion energy is defined as the energy difference between the GS ethylene structure (denoted as planar ethylene) and the orthogonally rotated ethylene structure (denoted as twisted ethylene), which are both shown in the inset of Figure 2. Here, we consider only the singlet states for both configurations. It was shown^59^ that the JSD ansatz cannot describe the torsion energy correctly since the ansatz cannot consider the static electronic correlation of the twisted ethylene, which has a diradical character. This is true both at the variational and at the FN level of theory.^59^ The lack of reliability in the FN results based on a JSD ansatz indicates that projection schemes cannot recover strong correlation if the FN constraints are given from a wave function with qualitative issues, due to the constraint on the projection coming from the trial wave function. Thus, the way to improve the quality of the FN results is to adopt a more general ansatz, able to improve the nodes of the trial wave function and enhance the reliability of FN estimations.

The planar ethylene has an electronic structure characterized by a highest occupied MO (HOMO) of type π and a lowest unoccupied MO (LUMO) of type π*, and the HOMO–LUMO gap is finite. A single SD having two electrons of unlike spin on the HOMO and no electrons on the LUMO captures qualitatively well the nature of the wave function and there is no static correlation. However, when the molecule is twisted, the HOMO–LUMO gap decreases because the overlap between the p orbitals (orthogonal to the plane of the −CH_2_ atoms) of the two carbons decreases. At a torsional angle of 90° (i.e., twisted ethylene), the two p orbitals become orthogonal and the frontier orbitals become degenerate, forming two singly occupied MOs. We can define three independent (orthogonal) wave functions having two electrons on two degenerate orbitals forming a spin singlet, a diradical, and two zwitterionic states.^75^ Their wave functions imply the use of more than one SD, i.e., their electronic structure shows strong correlation. Thus, a multireference ansatz is needed to correctly describe the diradical character of the orthogonally twisted ethylene.^59^

Figure 2 shows the torsion energies of ethylene computed with the JSD ansatz with an HF nodal surface and the same energies computed with the JAGPn ansatz with HF MOs,^g^ whose weights are optimized using DMC gradients. As a comparison, we also show results obtained with the full JAGP ansatz optimized using VMC gradients, which was taken from ref (59). The reference value in Figure 2 is taken from ref (76), and it is computed using MR-CISD + Q.^h^ The JSD ansatz gives 133.1(4) kcal/mol for the torsion energy, which is far from the reference value obtained by MR-CISD + Q (i.e., 69.2 kcal/mol^76^). Our JAGPn ansatz gives an FN energy of 73.0(4) kcal/mol for the torsion energy, which is close to the reference values. This result demonstrates that the JAGPn ansatz optimized using FN gradients correctly describes the diradical character of the orthogonally twisted ethylene, something that the JSD ansatz cannot do.

### 4.2 Application of FNAGPAS to Small and Large Systems

**Figure 3:**
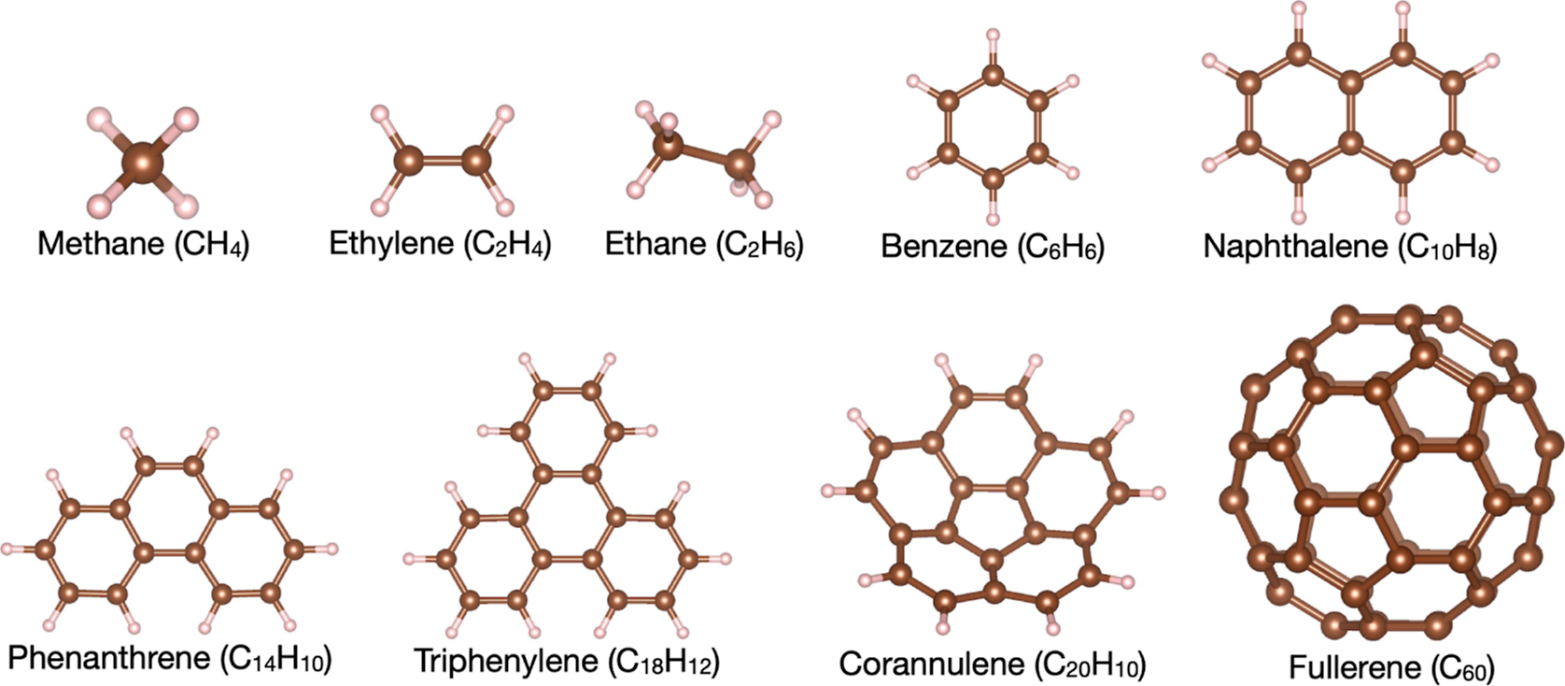
Molecular systems considered in this work, whose FN energy has been computed with the traditional JSD ansatz and with the JAGPn ansatz (within the FNAGPAS scheme) discussed in this work. The energy gain (i.e., the improvement of the FNAGPAS scheme over the traditional scheme which employs the JSD ansatz) and the number of variational parameters in the wave function for each system are shown in Figure 4.

We now show that the FNAGPAS scheme leads to a systematic improvement over the traditional JSD ansatz in molecular systems of increasing size, showing an accuracy in line with the full JAGP ansatz (and better on systems where the optimization error for the JAGP ansatz is large), while being affordable on much larger systems. We consider the eight hydrocarbons and the C_60_ fullerene, represented in Figure 3.

**Figure 4:**
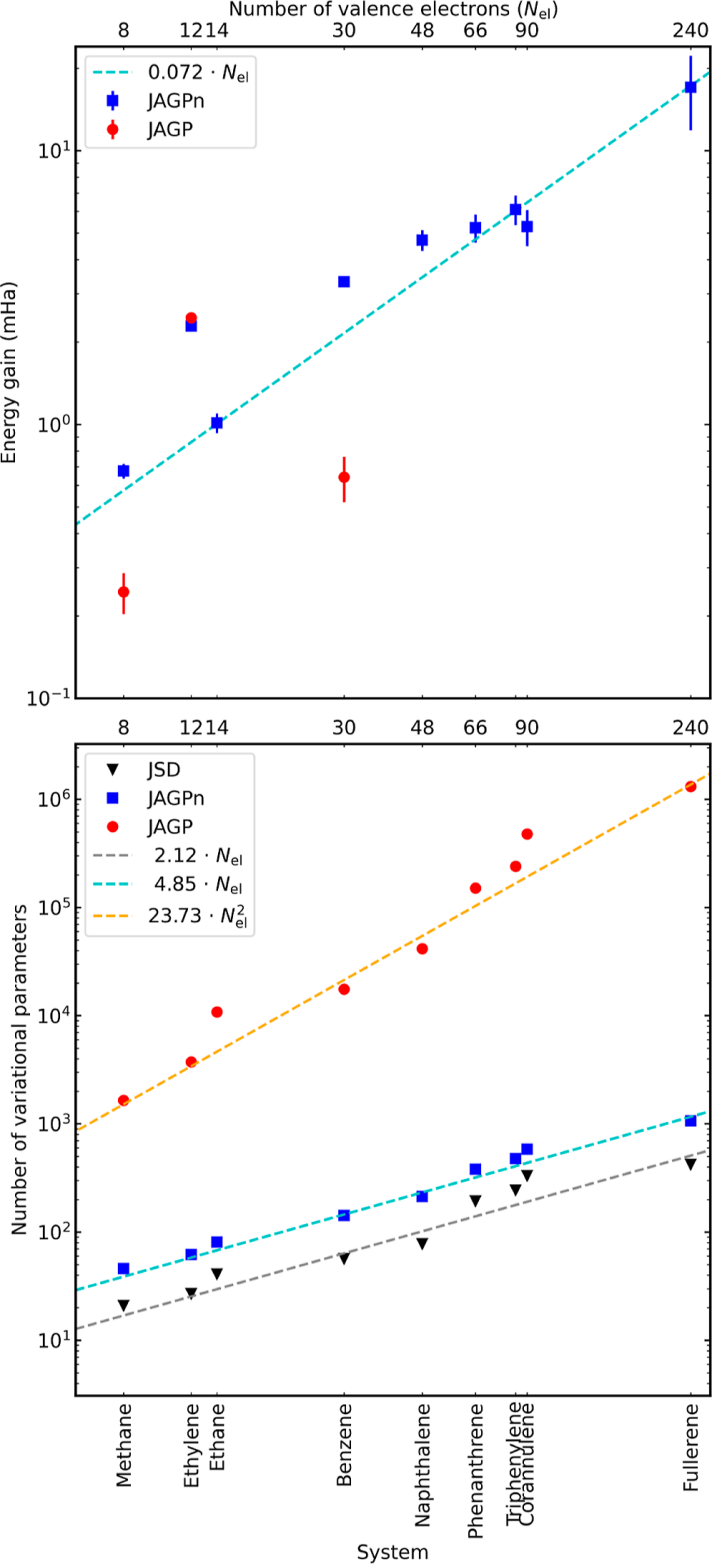
Top panel shows the improvement, dubbed energy gain, of the JAGP (red) and JAGPn (blue) ansatz with respect to the traditional JSD ansatz for each of the considered systems, as a function of the number of valence electrons. The energy gain is the difference between FN energy of the JSD ansatz and the JAGP (or JAGPn) ansatz. The bottom panel shows the number of parameters in the Jastrow factor, in the JAGP, and in the JAGPn wave functions. The dashed lines show the linear (gray for JSD and cyan for JAGPn) and quadratic (orange for JAGP) fitting curves.

Figure 4 (top panel) shows the energy gain in the LRDMC total energies (*a* → 0) by the nodal-surface optimizations of JAGP and JAGPn over the traditional JSD ansatz (with the nodal surface taken from the DFT LDA calculations). Our proposed FNAGPAS scheme (JAGPn ansatz optimized using FN gradients) shows positive gains for all molecules, indicating that the nodal-surface optimizations improve the nodes of the SD obtained from DFT. Therefore, there is a systematic improvement in the description of the correlation energy. The energy gain scales linearly with the number of electrons in the system. The traditional JAGP ansatz (optimized using VMC gradients) was computationally affordable only on the four smallest systems, due to the rapid increase of the number of variational parameters (see the bottom panel in Figure 4), which makes the optimization unstable or not converging. In addition, we could only use VMC-opt for the JAGP ansatz because FN-opt is not stable. This highlights an additional crucial advantage of FNAGPAS over the traditional JAGP approach. In the four systems where we have both the traditional JAGP and the FNAGPAS results, the latter is equivalent to the former on ethane, and it recovers more correlation energy in methane, ethylene, and benzene. Larger systems were computationally unaffordable with JAGP, while JAGPn optimization remains feasible both at the variational and at the FN level. In fact, FNAGPAS has been successfully performed up to C_60_ fullerene. The gain in C_60_ is ∼2 meV/valence electron, as shown in the inset of Figure 4. This is a reasonable value, considering a previous study by Marchi et al. reporting ∼3 meV/valence electron for the finite-size graphene calculations with the same atoms as the C_60_.^77^

Let us consider more closely the medium-size molecules. Figure 4 shows that the gains of JAGPn (optimized with FN gradients) are larger than those of JAGP (optimized using VMC gradients) in spite of the compactness of the AGPn ansatz. In fact, the number of variational parameters in the benzene molecule is 86 for the JAGPn ansatz and is 17,629 for the JAGP ansatz. Moreover, JAGP is a generalization of JAGPn. Therefore, one could naively expect that the larger the number of variational parameters, the lower the energy. Here, we observe an exception to this expectation. For this point, we recall that the calculations reported in Figure 4 are obtained with a quite small Jastrow factor, employing a [3s1p] basis set for C atoms and a [3s] for H atoms. This is because we target large systems with FNAGPAS, for which the use of large Jastrow factors is unaffordable. It has been reported that an incomplete Jastrow factor leads to misdirection of the nodal surface within the variational optimization of the JAGP ansatz in the square H_4_.^78^ To confirm if this is the case in the present calculations, we performed additional calculations with a larger Jastrow factor in the JAGP ansatz calculations (i.e., a basis set of [4s3p1d] and [3s1p] for C and H atoms, respectively) and obtained that the larger Jastrow factor leads to a much larger energy gain than that obtained with the JAGP ansatz with a small Jastrow [see results in the Supporting Information (Table S-I and Figure S-I)]. The result indicates that the small Jastrow factor leads to misdirection of the nodal surface of the JAGP ansatz also in this study. On the other hand, Figure 4 demonstrates that the FNAGPAS scheme works even with a small Jastrow factor and a minimal number of parameters in the antisymmetric part, making the approach applicable to larger systems.

As mentioned in the method part, see Section 2, the two main features over which FNAGPAS is built are (1) the AGPn ansatz and (2) the optimization of its nodal surface using FN gradients. To reveal which of the two is more crucial for the success of the method, i.e., the ansatz or the gradient, we tried the following combinations: (i) JAGPn with VMC-opt; (ii) JAGPn with FN-opt, (iii) JAGP with VMC-opt; and (iv) JAGP with FN-opt. Note that (ii) corresponds to FNAGPAS. The scheme (iv), unfortunately, is not possible as the JAGP has too many parameters and the FN optimization becomes unstable. Results obtained with schemes (i–iii) are reported in the Supporting Information (Table S-I and Figure S-I). We observe that scheme (ii) gives the best gains. Scheme (i) gives gains close to (ii), and they both are much better than (iii). Thus, it appears that freezing the orbitals to those obtained by a mean-field approach plays a crucial role in avoiding misdirection of the node optimization.

### 4.3 FNAGPAS Scheme Is Size-Consistent

We have shown that the AGPn ansatz is able to gain correlation energies at the FN level using very few variational parameters. In addition to their role in improving the nodal surface, FN gradients also appear to be crucial when calculating binding energies of molecules, preserving size consistency. As shown in Table 1 and discussed hereafter for the particular case of the water–methane dimer, this is not the case when VMC gradients are used. Therefore, when calculating binding energies of molecules, the use of VMC gradients in the JAGPn ansatz gives incorrect results, while the use of FN gradients plays a crucial role in it.

**Table 1:** FN Binding Energy *E*_b_ and Size-Consistency Energy Error *E*_SCE_, Computed with LRDMC *a* → 0, as Obtained with the JSD, JAGPn, and JAGP Wave Functions^a^

ansatz	nodes opt	*E*_b_ (meV)	*E*_SCE_ (meV)
JSD		–27(2)	–1(1)
JAGPn	VMCopt	–46(2)	10(2)
JAGPn	FNopt	–29(2)	–2(2)
JAGP	VMCopt	–41(3)	11(3)
CCSD(T)		–27	0

a For JAGPn, we consider both the case of using VMC and FN gradients to optimize the nodal surface. The latter is the scheme dubbed FNAGPAS in this work.

Table 1 contains the binding energies of the methane–water dimer computed with the JSD ansatz, with the JAGPn ansatz optimized using either VMC or FN gradients (the FNAGPAS approach), and with the JAGP ansatz optimized with VMC gradients. The binding energy is evaluated as the energy difference between the dimer and the sum of the energies of the two molecules: *E*_b_ = *E*_water–methane_ – *E*_water_ – *E*_methane_. The reference value for the binding energy of the water–methane dimer, −27 meV, was computed by CCSD(T) implemented in the Orca(79,80) program.^i^ We chose the CCSD(T) value as a reference because the bounded water–methane dimer is not a strongly correlated system, thus CCSD(T) should describe the binding energy correctly. In this system, the JSD ansatz gives a binding energy of −27(2) meV, which is in good agreement with the CCSD(T) values of −27.0 meV. Thus, a new DMC approach with nodal-surface optimization should lower the value of the total energies but should not affect the energy differences. The FNAGPAS scheme, which optimizes the JAGPn parameters with the FN gradients, behaves as expected, yielding a binding energy of −29(2) meV, still in good agreement with the reference value. However, this is not the case for the JAGPn ansatz optimized with the VMC gradients, which gives *E*_b_ = −46(2) meV, or for the JAGP ansatz (with VMC optimization), which gives *E*_b_ = −41(3) meV.

We can interpret the deterioration of the binding energy as follows: binding energies are computed from relative energies among two or more molecules; thus, the accuracy relies on its error cancellation. The error cancellation in DMC was reviewed and discussed by Dubecký in 2016.^8^ Their conclusion is that one can rely on error cancellation as long as one keeps the constructions and optimizations of the corresponding wave functions as systematic as possible. Indeed, this cancellation works when the nodes are kept at the same systematic accuracy at every step of the trial wave function constructions. In fact, for the water–methane dimer calculations in this study, our JSD ansatz fully satisfies the size consistency and gives satisfactory binding energy, which means that the error cancellation works with the DFT nodal surfaces. In this study, we found that error cancellation was deteriorated by the nodal-surface optimizations using the VMC gradients while recovered by those using the FN-DMC gradients. When one computes the binding energy of a complex system, one usually uses the same Jastrow basis sets for each element in the complex and the isolated systems. The use of the same Jastrow basis sets does not guarantee the same contribution to the total energy of both the complex and the isolated systems at the VMC level. Indeed, during the nodal-surface optimization at the VMC level, the incomplete Jastrow factor affects the nodal surface differently between the complex and isolated systems; thus, the resultant nodal surface gives the incorrect binding energy. The recovery should be because FN-DMC is a projection method to relax the amplitude of the AGPn ansatz, which corresponds to adding an unlimited flexible Jastrow factor to a given ansatz.

The Jastrow incompleteness is also related to the deterioration of the size consistency for JAGPn and JAGP with VMC optimization. The size consistency is a property that guarantees the consistency of the energy behavior when the interaction between the involved molecular system is nullified (e.g., by a long distance). If the size consistency is fulfilled, the energy of the far-away system should be equal to the sum of the energies of the two isolated molecules. The last column in Table 1 shows the difference in energies of the far-away water–methane complex (at a distance of ∼11 Å) and the sum of the isolated molecules, which can be considered the size-consistency error and is here dubbed *E*_SCE_. The JSD ansatz is size-consistent, as expected.^81^ The table clearly shows that the size consistency is deteriorated by the optimization using VMC gradients, i.e., the difference between the isolated and far-away energies is finite. In contrast, the size consistency is perfectly retrieved by the optimization using FN gradients. Neuscamman^63^ pointed out that the deterioration of the size consistency comes from an incomplete Jastrow factor. More specifically, the real-space three/four-body Jastrow factor, which was employed in the present study, cannot completely remove the size-consistency error unless we use unlimited flexibility in the Jastrow.^63^ To solve the problem, Goetz and Neuscamman proposed the so-called number-counting Jastrow factors that can suppress the unfavorable ionic terms and is able to solve the size-consistency problem^82,83^ within the VMC framework. In this regard, our proposed scheme can be interpreted as an alternative approach because, again, FN-DMC is a projection method to relax the amplitude of the AGPn ansatz, which corresponds to adding an unlimited flexible Jastrow factor to a given ansatz.

### 4.4 Discussion

First, we compare our approach with others that also target to go beyond the single-reference FN approximation. A well-established strategy is to use the multideterminant ansatz, which has witnessed numerous successes so far.^84−92^ The multideterminant approach offers the advantage of systematic improvement by increasing the number of SDs. Nonetheless, the number of SDs for a comprehensive representation exponentially scales with system size, imposing substantial computational demands for large systems. Therefore, this method has mainly been applied to small molecular systems.^84−86^ However, there have been successful efforts to reduce the number of required determinants by neglecting less important ones^87,88^ using, for instance, the configuration interaction using a perturbative selection made iteratively (CIPSI), which mitigates the exponential character of the multideterminant approach in practice.^90,92^ Recently, Benali et al. successfully applied the multideterminant approach for solids with more than a hundred electrons by combining the CIPSI technique with a restricted active space built using NOs,^91^ which is a similar idea as we present in this study. Indeed, they demonstrated that one can go beyond the single-reference nodal surface in large systems by the multideterminant approach in practice, though its naive asymptotic scaling is exponential. The multideterminant approach is becoming as practical and promising as the single-determinant approach.

Concerning the actual computational costs of our proposed methods, the choice of ansatz (i.e., JSD or AGPn) does not significantly affect the cost of wave function optimization, while the choice of gradients does. For instance, for C_60_, Jastrow optimization with the JSD ansatz and Jastrow + nodal-surface (i.e., weights of NOs) optimization with the JAGPn ansatz using VMC gradients require 11.9 and 43.6 core hours per optimization step with ∼7 mHa accuracy on the total energy evaluation at each optimization step, respectively.^j^ However, if one uses FN gradients for wave function optimization, one needs more computational time. For instance, for C_60_, the nodal-surface (i.e., weights of NOs) optimization with the JAGPn ansatz using FN gradients with *a* = 0.20 au requires 195.3 core hours per optimization step with ∼7 mHa accuracy on the total energy evaluation at each optimization step. Thus, our FNAGPAS scheme using FN gradients shows the same scaling of the number of variational parameters as the single-reference FN DMC with JSD ansatz, while it increases the prefactor of computational cost.

Based on the results obtained in this work so far, we finally discuss how to improve a Fermionic ansatz in ab initio QMC calculations, in general. Recently, there have been many successful reports about machine-learning-inspired ansatz with a huge degree of freedom in describing electronic and spin states, such as deep neural networks,^93^ restricted Boltzmann machines,^94−96^ and transformers,^97^ which are utilized as ansatz of wave functions to solve the Schrödinger equation with lattice Hamiltonians. Also, in the ab initio community, ansatzes using deep neural networks have been successfully applied for realistic problems, such as PauliNet,^42^ FermiNet,^39^ and others.^40,41,43−45^ In light of the present results, let us consider exploiting an ansatz with a huge degree of freedom (i.e., many variational parameters) in ab initio QMC calculations to pursue an exact Fermionic GS. If we stop at the VMC level, we may apply such a flexible ansatz to Jastrow factors, the determinant part, or both parts, and it is expected that the larger the degree of freedom an ansatz has, the larger the energy gain should be. However, improvements at the VMC level do not necessarily lead to improvements at the FN level, especially if the determinant part is optimized at the variational level. A variational optimization improves the overall shape of the trial wave function Ψ_T_, while the nodal surface might not be as optimized as the Ψ_T_. In this work, indeed, we have seen how the JAGPn ansatz optimized at the FN level leads to much better results than the JAGP ansatz optimized at the VMC level, despite the latter having many more variational parameters and it is much better at the VMC level. Moreover, we also observed how the JAGPn (and JAGP, for that matter) ansatz itself yields a size-consistency error at the FN level if the parameters are optimized at the VMC level, while the same ansatz with parameters optimized at the FN level is not affected by this issue. Thus, caution should be used when employing these new highly flexible machine-learning-based wave function parameterizations, as it is not guaranteed that improvements in the VMC energy are reflected in improvements in the FN energy in a consistent way. Basic physical properties, which were present in the most standard wave functions (such as the JSD), might not appear in the fancier approaches, similar to the mentioned problem of size inconsistency in JAGP and JAGPn.

## 5 Conclusions and Perspectives

In this study, we propose a method for variational optimization of the AGP wave function expressed in terms of NOs, with pairing coefficients optimized using FN gradients. Within our scheme, the variational parameter space increases only linearly with the system size, as opposed to the quadratic scaling of the standard parameterization of AGP, with the result that our proposed method allows the optimization of the nodal surfaces for large systems, which has been difficult to achieve with conventional approaches. In addition to demonstrating that our scheme can be applied to systems as large as C_60_, we showed that our scheme also achieves better (i.e., lower) DMC energies than the single-reference FN DMC calculations. Moreover, we have shown that our approach is size-consistent and can be used to estimate binding energies.

We showed that the Jastrow incompleteness affecting nodal-surface optimizations can be mitigated by using FN gradients combined with the JAGPn ansatz. However, in this study, we did not investigate the effect of the basis set incompleteness on the determinant part (i.e., nodal surface). The basis set incompleteness is believed to be less severe in QMC calculations than in quantum chemistry methods because the Jastrow factor (at the variational level) or the projection (at the FN level) mitigates its error. However, to the best of our knowledge, no one has seriously investigated the error so far. Considering binding energy calculations done by DMC reported so far,^8^ the basis set incompleteness should have a small effect on small molecules, but it should be carefully considered when studying large molecules using DMC done with localized basis sets. This is one of the intriguing future works for applying the single-reference DMC and our proposed methods to large systems.

## Data Availability

The QMC kernel used in this work, TurboRVB, is available from its GitHub repository [https://github.com/sissaschool/turborvb].

## Appendix

### Proof for the Variational Principle of the LRDMC Optimization with DLA

As pointed out in seminal works by Casula et al.,^72,98^ the use of a pseudopotential that has the so-called nonlocal term induces an additional sign problem in the standard DMC approach with the LA; thus the variational principle, which justifies the energy minimization strategy, is deteriorated. Instead, one of the advantages of the LRDMC is that the use of pseudopotentials does not deteriorate the variational principle;^72^ thus, the energy minimization is justified. Recently, we implemented the DLA^18^ into the TurboRVB package. In this study, we combine the DLA with the LRDMC framework implemented in the TurboRVB package. We prove here that the variational principle holds also in the LRDMC with the DLA. This proof is inspired by the proof by Haaf et al.^99^ that the lattice Green’s function Monte Carlo method is variational.

In LRDMC calculations with the DLA, the effective Hamiltonian (i.e., the FN Hamiltonian) reads

4

where , , by which the original term in the LRDMC approach, , is replaced, and sf means that all *x*′ (≠*x*) satisfy Ψ_T_(*x*′)*H*_*x*,*x*′_Ψ_T_(*x*) > 0. Here, we omit the lattice-space dependency of the Hamiltonian (i.e., *H* ≡ *H*^*a*^) because one can extrapolate energies to the *a* → 0 limit. Notice that we assume that a trial wave function can be decomposed into the Jastrow and determinant parts, i.e., Ψ_T_ = *J*_T_*D*_T_. We also notice that

since the Jastrow factor does not affect the sign of a wave function. We define the following notations:

5

6

7

8

where |Φ_FN_⟩ is the FN GS of *Ĥ*^FN^. In the following, we will show that the following equations hold:

9

The first equality (*E*_MA_ = *E*_FN_) holds because |Φ_FN_⟩ is the exact GS of *H*^FN^ (i.e., *Ĥ*^FN^|Φ_FN_⟩ = *E*_FN_|Φ_FN_⟩). This is also true with the nonlocal terms of pseudopotentials. Now, we define the difference between the effective FN energy obtained with the effective Hamiltonian *Ĥ*^FN^ and that obtained with the true Hamiltonian *Ĥ*:

10

We want to prove that Δ*E* ≥ 0 for the FN state and that the equality holds for Φ_FN_ = Ψ_T_ = Ψ_0_, where we denote Ψ_0_ as the exact wave function of the original Hamiltonian *Ĥ*, i.e., . Hereafter, we will do the same exercise written in ref (99). We define the difference between the effective FN energy obtained with the effective Hamiltonian *H*^FN^ and that obtained with the true Hamiltonian *H*:

11

where we define a truncated Hamiltonian *H*^tr^ and a spin-flip Hamiltonian *H*^sf^ by

12

and

13

Indeed, the matrix elements are

14

and

15

Δ*E* can be written explicitly in terms of the matrix elements:

16

This can be rewritten as

17

where sf means that all *x*′ (≠ *x*) satisfy Ψ_T_(*x*′)*H*_*x*,*x*′_Ψ_T_(*x*) > 0. In this double summation, each pair of configurations (*x*, *x*′) appears twice. Therefore, we can combine these terms and rewrite it as a summation over the pairs:

18

Notice that the Hamiltonian is Hermitian: *H*_*x*,*x*′_ = *H*_*x*′,*x*_. Since all the pairs satisfy , we obtain

19

Then

20

where sgn(*x*, *x*′) denotes the sign of *H*_*x*,*x*′_. Finally, we get

21

indicating that Δ*E* is positive for any wave function Φ_FN_. Thus, the GS energy of *H*^FN^ is an upper bound for the GS energy of the original Hamiltonian *H* (i.e., *E*_FN_ ≥ *E*). Hereafter, we consider the case that one uses the true GS Ψ_0_ for the determinant of the trial wave function (i.e., Ψ_T_ = *J*_T_·Ψ_0_), to prove that *E*_FN_ = *E* holds with Ψ_T_ = *J*_T_·Ψ_0_ (i.e., *D*_T_ = Ψ_0_): For all the pairs (*x*, *x*′), Ψ_0_*H*_*x*,*x*′_Ψ_0_ > 0 is satisfied, meaning sgn(*x*, *x*′) → + and  or sgn(*x*, *x*′) → – and . Thus, the above condition is fulfilled when the following condition is satisfied:

22

In the DLA approach, the spin-flip term is composed only of the determinant of the trial wave function. Therefore, the FN outcome with the DLA approach is not affected by the presence of the Jastrow factor in the trial wave function (in the *a* → 0 limit). Therefore, one gets Φ_FN_ = Ψ_0_ with Ψ_T_ = *J*_T_·Ψ_0_. Thus, Δ*E* = 0 is fulfilled with Ψ_T_ = *J*_T_·Ψ_0_, and the following relations hold:

23

meaning that the effective Hamiltonian  and the true Hamiltonian *Ĥ* have the same GS energy *E*_0_ and the same GS Φ_FN_ = Ψ_0_ with Ψ_T_ = *J*_T_ ·Ψ_0_, where the final equality *E* = *E*_0_ comes from the usual variational principle.

In the DLA approach, we can update the trial wave function Ψ_T_ such that *E*_MA_ goes down according to the gradient ∂_α_*E*_MA_ or using a more sophisticated optimization scheme. As written above, the equals *E*_FN_ = *E* = *E*_0_ are met when Ψ_T_ = *J* ·Ψ_0_. It implies that one can look for the true GS energy and wave function by variation of the determinant part of the trial wave function. Indeed, in the LRDMC calculations with the DLA, one can access the mixed-average energy *E*_MA_ and its derivative , where α⃗ is a set of the variational parameters. Since *E*_MA_ satisfies the variational principle, i.e., *E*_MA_ ≥ *E*_0_, the equality holds when Ψ_T_ = *J*_T_·Ψ_0_; as proven above, one can update the determinant part of the trial wave function, *D*_T_, such that *E*_MA_ goes down, then, it is expected that *D*_T_ finally reaches *D*_T_ → Ψ_0_ and *E*_MA_ → *E*_0_.

## Supporting Information Available

Total energies and the number of variational parameters of hydrocarbons and fullerene, total energies of the methane–water complex and corresponding fragments and of the planar and twisted ethylene molecule, and discussion on the role of MOs or NOs in the AGPn ansatz and the size-consistency error (PDF)
Geometries, in xyz format, of all the systems studied (ZIP)

## Footnotes

a In all-electron calculations, FN-DMC is always variational, meaning that the lowest FN energy *E*_FN_ is obtained when the exact nodal surface is used, otherwise *E*_FN_ > *E*_0_. When pseudopotentials are employed, there are also nonlocal operators in the Hamiltonian. This yields to a problem similar to the sign problem, which requires a further approximation. There are a few alternatives to deal with pseudopotentials in DMC: the LA,^15^ the T-move (TM),^16,17^ the DLA, and the determinant locality TM (DLTM).^18^ TM and DLTM are variational, meaning that their energy (*E*_FN,TM_ or *E*_FN,DLTM_) is an upper bound of the exact GS energy *E*_0_.

b The Jastrow factor correlates explicitly all pairs of electrons; it is a symmetric positive function, so it recovers dynamical correlation and it does not change the nodal surface.

c An approach is size-consistent if the energy of a system constituted by two or more noninteracting subsystems (e.g., two molecules far away) is the same of the sum of the energies of the subsystems.

d See ref (57) for details about the functional form of terms implemented in the TurboRVB package used for this work.

e It is generally claimed that the cost of FN-DMC scales as the cube of the number of electrons *N*_el_. This is true for simulations where the antisymmetric part of the wave function can be computed as a determinant and *N*_el_ up to roughly a thousand. For larger systems, the cost for a MC step is  and therefore the cost of FN-DMC is quartic.

f The use of DLA in LRDMC is equivalent to the DLTM^18^ scheme in standard DMC.

g The HF orbitals obtained with the Fermi–Dirac smearing method were used for the occupied and the virtual orbitals of the JAGPn ansatz for the twisted ethylene because the HOMO and LUMO should have the same energies. Note, in this case, we did not use the NOs (introduced in the discussion above) because this system is characterized by strong correlation coming from the two frontier orbitals, which are easily derived already from the HF theory. Moreover, in the twisted ethylene case, we allowed the optimization of the off-diagonal coefficient of the AGP matrix that pairs the two frontier orbitals.

h The twisted ethylene is a prototypical example of a system characterized by strong correlation where single-reference perturbative approaches, such as CCSD(T), fail and multireference approaches are needed.

i In particular, we performed canonical CCSD(T) calculations with the automatic basis set extrapolation implemented in Orca (which assumes an exponential convergence for the HF energy and a polynomial convergence for the correlation energy) using Dunning correlation-consistent core-polarized basis sets, cc-pCV*n*Z, with quadruple-ζ (*n* = Q) and quintuple-ζ (*n* = 5) basis sets. We performed both estimations with and without counterpoise correction, both yielding a binding energy of −27.2 meV.

j We measured the computational times on the Numerical Materials Simulator at the National Institute for Materials Science (NIMS) using 1536 cores [32 nodes × Intel Xeon Platinum 8268 (2.9 GHz, 24 cores) × 2 per node].

## References

[ref1] Hohenberg P.; Kohn W. Inhomogeneous Electron Gas. Phys. Rev. 1964, 136, B864–B871. 10.1103/PhysRev.136.B864.

[ref2] Bartlett R. J.; Musiał M. Coupled-cluster theory in quantum chemistry. Rev. Mod. Phys. 2007, 79, 291–352. 10.1103/RevModPhys.79.291.

[ref3] Řezáč J.; Hobza P. Describing Noncovalent Interactions beyond the Common Approximations: How Accurate Is the “Gold Standard,” CCSD(T) at the Complete Basis Set Limit?. J. Chem. Theory Comput. 2013, 9, 2151–2155. 10.1021/ct400057w. 26583708

[ref4] Řezáč J.; Hobza P. Benchmark Calculations of Interaction Energies in Noncovalent Complexes and Their Applications. Chem. Rev. 2016, 116, 5038–5071. 10.1021/acs.chemrev.5b00526. 26943241

[ref5] Al-Hamdani Y. S.; Tkatchenko A. Understanding non-covalent interactions in larger molecular complexes from first principles. J. Chem. Phys. 2019, 150, 010901 10.1063/1.5075487. 30621423 PMC6910608

[ref6] Szalay P. G.; Müller T.; Gidofalvi G.; Lischka H.; Shepard R. Multiconfiguration Self-Consistent Field and Multireference Configuration Interaction Methods and Applications. Chem. Rev. 2012, 112, 108–181. 10.1021/cr200137a. 22204633

[ref7] Foulkes W. M. C.; Mitas L.; Needs R. J.; Rajagopal G. Quantum Monte Carlo simulations of solids. Rev. Mod. Phys. 2001, 73, 33–83. 10.1103/RevModPhys.73.33.

[ref8] Dubecký M.; Mitas L.; Jurečka P. Noncovalent Interactions by Quantum Monte Carlo. Chem. Rev. 2016, 116, 5188–5215. 10.1021/acs.chemrev.5b00577. 27081724

[ref9] Al-Hamdani Y. S.; Rossi M.; Alfè D.; Tsatsoulis T.; Ramberger B.; Brandenburg J. G.; Zen A.; Kresse G.; Grüneis A.; Tkatchenko A.; Michaelides A. Properties of the water to boron nitride interaction: from zero to two dimensions with benchmark accuracy. J. Chem. Phys. 2017, 147, 044710 10.1063/1.4985878. 28764374

[ref10] Brandenburg J. G.; Zen A.; Fitzner M.; Ramberger B.; Kresse G.; Tsatsoulis T.; Grüneis A.; Michaelides A.; Alfè D. Physisorption of Water on Graphene: Subchemical Accuracy from Many-Body Electronic Structure Methods. J. Phys. Chem. Lett. 2019, 10, 358–368. 10.1021/acs.jpclett.8b03679. 30615460

[ref11] Zen A.; Brandenburg J. G.; Klimeš J.; Tkatchenko A.; Alfè D.; Michaelides A. Fast and accurate quantum Monte Carlo for molecular crystals. Proc. Natl. Acad. Sci. U.S.A. 2018, 115, 1724–1729. 10.1073/pnas.1715434115. 29432177 PMC5828600

[ref12] Al-Hamdani Y. S.; Nagy P. R.; Zen A.; Barton D.; Kállay M.; Brandenburg J. G.; Tkatchenko A. Interactions between large molecules pose a puzzle for reference quantum mechanical methods. Nat. Commun. 2021, 12, 3927 10.1038/s41467-021-24119-3. 34168142 PMC8225865

[ref13] Shi B. X.; Zen A.; Kapil V.; Nagy P. R.; Grüneis A.; Michaelides A. Many-Body Methods for Surface Chemistry Come of Age: Achieving Consensus with Experiments. J. Am. Chem. Soc. 2023, 145, 25372–25381. 10.1021/jacs.3c09616. 37948071 PMC10683001

[ref14] Troyer M.; Wiese U. J. Computational complexity and fundamental limitations to fermionic quantum Monte Carlo simulations. Phys. Rev. Lett. 2005, 94, 170201 10.1103/PhysRevLett.94.170201. 15904269

[ref15] Mitas L.; Shirley E. L.; Ceperley D. M. Nonlocal pseudopotentials and diffusion Monte Carlo. J. Chem. Phys. 1991, 95, 3467–3475. 10.1063/1.460849.

[ref16] Casula M. Beyond the locality approximation in the standard diffusion Monte Carlo method. Phys. Rev. B: Condens. Matter Mater. Phys. 2006, 74, 161102 10.1103/PhysRevB.74.161102.

[ref17] Casula M.; Moroni S.; Sorella S.; Filippi C. Size-consistent variational approaches to nonlocal pseudopotentials: Standard and lattice regularized diffusion Monte Carlo methods revisited. J. Chem. Phys. 2010, 132, 154113 10.1063/1.3380831. 20423174

[ref18] Zen A.; Brandenburg J. G.; Michaelides A.; Alfè D. A new scheme for fixed node diffusion quantum Monte Carlo with pseudopotentials: Improving reproducibility and reducing the trial-wave-function bias. J. Chem. Phys. 2019, 151, 134105 10.1063/1.5119729. 31594339

[ref19] Casula M.; Sorella S. Geminal wave functions with Jastrow correlation: A first application to atoms. J. Chem. Phys. 2003, 119, 6500–6511. 10.1063/1.1604379.

[ref20] Casula M.; Attaccalite C.; Sorella S. Correlated geminal wave function for molecules: An efficient resonating valence bond approach. J. Chem. Phys. 2004, 121, 7110–7126. 10.1063/1.1794632. 15473777

[ref21] Marchi M.; Azadi S.; Casula M.; Sorella S. Resonating valence bond wave function with molecular orbitals: Application to first-row molecules. J. Chem. Phys. 2009, 131, 154116 10.1063/1.3249966. 20568856

[ref22] Bajdich M.; Mitas L.; Drobný G.; Wagner L. K.; Schmidt K. E. Pfaffian Pairing Wave Functions in Electronic-Structure Quantum Monte Carlo Simulations. Phys. Rev. Lett. 2006, 96, 130201 10.1103/PhysRevLett.96.130201. 16711968

[ref23] Bajdich M.; Mitas L.; Wagner L. K.; Schmidt K. E. Pfaffian pairing and backflow wavefunctions for electronic structure quantum Monte Carlo methods. Phys. Rev. B: Condens. Matter Mater. Phys. 2008, 77, 115112 10.1103/PhysRevB.77.115112.

[ref24] Genovese C.; Shirakawa T.; Nakano K.; Sorella S. General Correlated Geminal Ansatz for Electronic Structure Calculations: Exploiting Pfaffians in Place of Determinants. J. Chem. Theory Comput. 2020, 16, 6114–6131. 10.1021/acs.jctc.0c00165. 32804497 PMC8011928

[ref25] Toulouse J.; Umrigar C. J. Full optimization of Jastrow–Slater wave functions with application to the first-row atoms and homonuclear diatomic molecules. J. Chem. Phys. 2008, 128, 174101 10.1063/1.2908237. 18465904

[ref26] Zimmerman P. M.; Toulouse J.; Zhang Z.; Musgrave C. B.; Umrigar C. J. Excited states of methylene from quantum Monte Carlo. J. Chem. Phys. 2009, 131, 124103 10.1063/1.3220671. 19791848

[ref27] Anderson A. G.; Goddard W. A. Generalized valence bond wave functions in quantum Monte Carlo. J. Chem. Phys. 2010, 132, 164110 10.1063/1.3377091. 20441261

[ref28] Braïda B.; Toulouse J.; Caffarel M.; Umrigar C. J. Quantum Monte Carlo with Jastrow-valence-bond wave functions. J. Chem. Phys. 2011, 134, 084108 10.1063/1.3555821. 21361528

[ref29] Drummond N. D.; Ríos P. L.; Ma A.; Trail J. R.; Spink G. G.; Towler M. D.; Needs R. J. Quantum Monte Carlo study of the Ne atom and the Ne^+^ ion. J. Chem. Phys. 2006, 124, 224104 10.1063/1.2204600. 16784260

[ref30] Ríos P. L.; Ma A.; Drummond N. D.; Towler M. D.; Needs R. J. Inhomogeneous backflow transformations in quantum Monte Carlo calculations. Phys. Rev. E 2006, 74, 066701 10.1103/PhysRevE.74.066701. 17280171

[ref31] Toulouse J.; Umrigar C. J. Optimization of quantum Monte Carlo wave functions by energy minimization. J. Chem. Phys. 2007, 126, 084102 10.1063/1.2437215. 17343435

[ref32] Seth P.; Ríos P. L.; Needs R. J. Quantum Monte Carlo study of the first-row atoms and ions. J. Chem. Phys. 2011, 134, 084105 10.1063/1.3554625. 21361525

[ref33] Clark B. K.; Morales M. A.; McMinis J.; Kim J.; Scuseria G. E. Computing the energy of a water molecule using multideterminants: A simple, efficient algorithm. J. Chem. Phys. 2011, 135, 244105 10.1063/1.3665391. 22225142

[ref34] Morales M. A.; McMinis J.; Clark B. K.; Kim J.; Scuseria G. E. Multideterminant Wave Functions in Quantum Monte Carlo. J. Chem. Theory Comput. 2012, 8, 2181–2188. 10.1021/ct3003404. 26588949

[ref35] Filippi C.; Assaraf R.; Moroni S. Simple formalism for efficient derivatives and multi-determinant expansions in quantum Monte Carlo. J. Chem. Phys. 2016, 144, 194105 10.1063/1.4948778. 27208934

[ref36] Scemama A.; Applencourt T.; Giner E.; Caffarel M. Quantum Monte Carlo with very large multideterminant wavefunctions. J. Comput. Chem. 2016, 37, 1866–1875. 10.1002/jcc.24382. 27302337

[ref37] Dash M.; Moroni S.; Scemama A.; Filippi C. Perturbatively Selected Configuration-Interaction Wave Functions for Efficient Geometry Optimization in Quantum Monte Carlo. J. Chem. Theory Comput. 2018, 14, 4176–4182. 10.1021/acs.jctc.8b00393. 29953810 PMC6096455

[ref38] Scemama A.; Giner E.; Benali A.; Loos P.-F. Taming the fixed-node error in diffusion Monte Carlo via range separation. J. Chem. Phys. 2020, 153, 174107 10.1063/5.0026324. 33167659

[ref39] Pfau D.; Spencer J. S.; Matthews A. G. D. G.; Foulkes W. M. C. Ab initio solution of the many-electron Schrödinger equation with deep neural networks. Phys. Rev. Res. 2020, 2, 033429 10.1103/PhysRevResearch.2.033429.

[ref40] Li X.; Fan C.; Ren W.; Chen J. Fermionic neural network with effective core potential. Phys. Rev. Res. 2022, 4, 013021 10.1103/PhysRevResearch.4.013021.

[ref41] Ren W.; Fu W.; Wu X.; Chen J. Towards the ground state of molecules via diffusion Monte Carlo on neural networks. Nat. Commun. 2023, 14, 1860 10.1038/s41467-023-37609-3. 37012248 PMC10070323

[ref42] Hermann J.; Schätzle Z.; Noé F. Deep-neural-network solution of the electronic Schrödinger equation. Nat. Chem. 2020, 12, 891–897. 10.1038/s41557-020-0544-y. 32968231

[ref43] Choo K.; Mezzacapo A.; Carleo G. Fermionic neural-network states for ab-initio electronic structure. Nat. Commun. 2020, 11, 2368 10.1038/s41467-020-15724-9. 32398658 PMC7217823

[ref44] Li R.; Ye H.; Jiang D.; Wen X.; Wang C.; Li Z.; Li X.; He D.; Chen J.; Ren W. others Forward Laplacian: A New Computational Framework for Neural Network-based Variational Monte Carlo. arXiv 2023, arXiv:2307.08214.

[ref45] von Glehn I.; Spencer J. S.; Pfau D. A self-attention ansatz for ab-initio quantum chemistry. arXiv 2022, arXiv:2211.13672.

[ref46] Sorella S.; Seki K.; Brovko O. O.; Shirakawa T.; Miyakoshi S.; Yunoki S.; Tosatti E. Correlation-Driven Dimerization and Topological Gap Opening in Isotropically Strained Graphene. Phys. Rev. Lett. 2018, 121, 066402 10.1103/PhysRevLett.121.066402. 30141665

[ref47] Nakano K.; Maezono R.; Sorella S. All-Electron Quantum Monte Carlo with Jastrow Single Determinant Ansatz: Application to the Sodium Dimer. J. Chem. Theory Comput. 2019, 15, 4044–4055. 10.1021/acs.jctc.9b00295. 31117480

[ref48] Genovese C.; Shirakawa T.; Nakano K.; Sorella S. General Correlated Geminal Ansatz for Electronic Structure Calculations: Exploiting Pfaffians in Place of Determinants. J. Chem. Theory Comput. 2020, 16, 6114–6131. 10.1021/acs.jctc.0c00165. 32804497 PMC8011928

[ref49] Ammar A.; Giner E.; Scemama A. Optimization of Large Determinant Expansions in Quantum Monte Carlo. J. Chem. Theory Comput. 2022, 18, 5325–5336. 10.1021/acs.jctc.2c00556. 35997484

[ref50] Raghav A.; Maezono R.; Hongo K.; Sorella S.; Nakano K. Toward Chemical Accuracy Using the Jastrow Correlated Antisymmetrized Geminal Power Ansatz. J. Chem. Theory Comput. 2023, 19, 2222–2229. 10.1021/acs.jctc.2c01141. 37014742 PMC10134432

[ref51] Reboredo F. A.; Hood R. Q.; Kent P. R. C. Self-healing diffusion quantum Monte Carlo algorithms: Direct reduction of the fermion sign error in electronic structure calculations. Phys. Rev. B: Condens. Matter Mater. Phys. 2009, 79, 195117 10.1103/PhysRevB.79.195117.

[ref52] Reboredo F. A. Systematic reduction of sign errors in many-body problems: Generalization of self-healing diffusion Monte Carlo to excited states. Phys. Rev. B: Condens. Matter Mater. Phys. 2009, 80, 125110 10.1103/PhysRevB.80.125110.

[ref53] Reboredo F. A.; Kim J. Generalizing the self-healing diffusion Monte Carlo approach to finite temperature: A path for the optimization of low-energy many-body bases. J. Chem. Phys. 2014, 140, 074103 10.1063/1.4861222. 24559334

[ref54] Bajdich M.; Tiago M. L.; Hood R. Q.; Kent P. R.; Reboredo F. A. Systematic reduction of sign errors in many-body calculations of atoms and molecules. Phys. Rev. Lett. 2010, 104, 193001 10.1103/physrevlett.104.193001. 20866961

[ref55] McFarland J.; Manousakis E. Gradient-descent optimization of fermion nodes in the diffusion Monte Carlo technique. Phys. Rev. A 2022, 105, 032815 10.1103/PhysRevA.105.032815.

[ref56] Zen A.; Coccia E.; Gozem S.; Olivucci M.; Guidoni L. Quantum Monte Carlo Treatment of the Charge Transfer and Diradical Electronic Character in a Retinal Chromophore Minimal Model. J. Chem. Theory Comput. 2015, 11, 992–1005. 10.1021/ct501122z. 25821414 PMC4357234

[ref57] Nakano K.; Attaccalite C.; Barborini M.; Capriotti L.; Casula M.; Coccia E.; Dagrada M.; Genovese C.; Luo Y.; Mazzola G.; Zen A.; Sorella S. TurboRVB: A many-body toolkit for ab initio electronic simulations by quantum Monte Carlo. J. Chem. Phys. 2020, 152, 204121 10.1063/5.0005037. 32486669

[ref58] Annaberdiyev A.; Melton C. A.; Bennett M. C.; Wang G.; Mitas L. Accurate Atomic Correlation and Total Energies for Correlation Consistent Effective Core Potentials. J. Chem. Theory Comput. 2020, 16, 1482–1502. 10.1021/acs.jctc.9b00962. 32027496

[ref59] Zen A.; Coccia E.; Luo Y.; Sorella S.; Guidoni L. Static and dynamical correlation in diradical molecules by quantum monte carlo using the jastrow antisymmetrized geminal power ansatz. J. Chem. Theory Comput. 2014, 10, 1048–1061. 10.1021/ct401008s. 26580182

[ref60] Casula M.; Sorella S. Improper s-wave symmetry of the electronic pairing in iron-based superconductors by first-principles calculations. Phys. Rev. B: Condens. Matter Mater. Phys. 2013, 88, 155125 10.1103/PhysRevB.88.155125.

[ref61] Becca F.; Sorella S. Quantum Monte Carlo Approaches for Correlated Systems; Cambridge University Press, 2017.

[ref62] Sorella S.; Casula M.; Rocca D. Weak binding between two aromatic rings: Feeling the van der Waals attraction by quantum Monte Carlo methods. J. Chem. Phys. 2007, 127, 014105 10.1063/1.2746035. 17627335

[ref63] Neuscamman E. Size Consistency Error in the Antisymmetric Geminal Power Wave Function can be Completely Removed. Phys. Rev. Lett. 2012, 109, 203001 10.1103/PhysRevLett.109.203001. 23215480

[ref64] Grüneis A.; Booth G. H.; Marsman M.; Spencer J.; Alavi A.; Kresse G. Natural orbitals for wave function based correlated calculations using a plane wave basis set. J. Chem. Theory Comput. 2011, 7, 2780–2785. 10.1021/ct200263g. 26605469

[ref65] Sun Q.; Berkelbach T. C.; Blunt N. S.; Booth G. H.; Guo S.; Li Z.; Liu J.; McClain J. D.; Sayfutyarova E. R.; Sharma S.; et al. PySCF: the Python-based simulations of chemistry framework. Wiley Interdiscip. Rev.: Comput. Mol. Sci. 2018, 8, e1340 10.1002/wcms.1340.

[ref66] Sun Q.; Zhang X.; Banerjee S.; Bao P.; Barbry M.; Blunt N. S.; Bogdanov N. A.; Booth G. H.; Chen J.; Cui Z. H.; Eriksen J. J.; Gao Y.; Guo S.; Hermann J.; Hermes M. R.; Koh K.; Koval P.; Lehtola S.; Li Z.; Liu J.; Mardirossian N.; McClain J. D.; Motta M.; Mussard B.; Pham H. Q.; Pulkin A.; Purwanto W.; Robinson P. J.; Ronca E.; Sayfutyarova E. R.; Scheurer M.; Schurkus H. F.; Smith J. E.; Sun C.; Sun S. N.; Upadhyay S.; Wagner L. K.; Wang X.; White A.; Whitfield J. D.; Williamson M. J.; Wouters S.; Yang J.; Yu J. M.; Zhu T.; Berkelbach T. C.; Sharma S.; Sokolov A. Y.; Chan G. K. L. Recent developments in the PySCF program package. J. Chem. Phys. 2020, 153, 024109 10.1063/5.0006074. 32668948

[ref67] Nakano K.; Kohulák O.; Raghav A.; Casula M.; Sorella S. TurboGenius: Python suite for high-throughput calculations of ab initio quantum Monte Carlo methods. J. Chem. Phys. 2023, 159, 224801 10.1063/5.0179003. 38078530

[ref68] Posenitskiy E.; Chilkuri V. G.; Ammar A.; Hapka M.; Pernal K.; Shinde R.; Borda E. J. L.; Filippi C.; Nakano K.; Kohulák O.; Sorella S.; de Oliveira Castro P.; Jalby W.; Ríos P. L.; Alavi A.; Scemama A. TREXIO: A file format and library for quantum chemistry. J. Chem. Phys. 2023, 158, 174801 10.1063/5.0148161. 37144717

[ref69] Bennett M. C.; Melton C. A.; Annaberdiyev A.; Wang G.; Shulenburger L.; Mitas L. A new generation of effective core potentials for correlated calculations. J. Chem. Phys. 2017, 147, 224106 10.1063/1.4995643. 29246065

[ref70] Sorella S. Green function Monte Carlo with stochastic reconfiguration. Phys. Rev. Lett. 1998, 80, 4558–4561. 10.1103/PhysRevLett.80.4558.

[ref71] Sorella S.; Casula M.; Rocca D. Weak binding between two aromatic rings: Feeling the van der Waals attraction by quantum Monte Carlo methods. J. Chem. Phys. 2007, 127, 014105 10.1063/1.2746035. 17627335

[ref72] Casula M.; Filippi C.; Sorella S. Diffusion Monte Carlo method with lattice regularization. Phys. Rev. Lett. 2005, 95, 100201 10.1103/PhysRevLett.95.100201. 16196912

[ref73] Nakano K.; Maezono R.; Sorella S. Speeding up ab initio diffusion Monte Carlo simulations by a smart lattice regularization. Phys. Rev. B 2020, 101, 155106 10.1103/PhysRevB.101.155106.

[ref74] Momma K.; Izumi F. VESTA3 for three-dimensional visualization of crystal, volumetric and morphology data. J. Appl. Crystallogr. 2011, 44, 1272–1276. 10.1107/S0021889811038970.

[ref75] Salem L.; Rowland C. The Electronic Properties of Diradicals. Angew Chem. Int. Ed. Engl. 1972, 11, 92–111. 10.1002/anie.197200921.

[ref76] Barbatti M.; Paier J.; Lischka H. Photochemistry of ethylene: A multireference configuration interaction investigation of the excited-state energy surfaces. J. Chem. Phys. 2004, 121, 11614–11624. 10.1063/1.1807378. 15634126

[ref77] Marchi M.; Azadi S.; Sorella S. Fate of the resonating valence bond in graphene. Phys. Rev. Lett. 2011, 107, 086807 10.1103/PhysRevLett.107.086807. 21929194

[ref78] Genovese C.; Meninno A.; Sorella S. Assessing the accuracy of the Jastrow antisymmetrized geminal power in the H_4_ model system. J. Chem. Phys. 2019, 150, 084102 10.1063/1.5081933. 30823772

[ref79] Neese F. The ORCA program system. Wiley Interdiscip. Rev.: Comput. Mol. Sci. 2012, 2, 73–78. 10.1002/wcms.81.

[ref80] Neese F. Software update: the ORCA program system, version 4.0. Wiley Interdiscip. Rev.: Comput. Mol. Sci. 2018, 8, 4–9. 10.1002/wcms.1327.

[ref81] Zen A.; Sorella S.; Gillan M. J.; Michaelides A.; Alfè D. Boosting the accuracy and speed of quantum Monte Carlo: Size consistency and time step. Phys. Rev. B 2016, 93, 241118 10.1103/physrevb.93.241118.

[ref82] Van Der Goetz B. W.; Neuscamman E. Suppressing Ionic Terms with Number-Counting Jastrow Factors in Real Space. J. Chem. Theory Comput. 2017, 13, 2035–2042. 10.1021/acs.jctc.7b00158. 28384404

[ref83] Van Der Goetz B. W.; Otis L.; Neuscamman E. Clean and Convenient Tessellations for Number Counting Jastrow Factors. J. Chem. Theory Comput. 2019, 15, 1102–1121. 10.1021/acs.jctc.8b01139. 30620589

[ref84] Booth G. H.; Alavi A. Approaching chemical accuracy using full configuration-interaction quantum Monte Carlo: A study of ionization potentials. J. Chem. Phys. 2010, 132, 174104 10.1063/1.3407895. 20459153

[ref85] Petruzielo F. R.; Toulouse J.; Umrigar C. J. Approaching chemical accuracy with quantum Monte Carlo. J. Chem. Phys. 2012, 136, 124116 10.1063/1.3697846. 22462844

[ref86] Morales M. A.; McMinis J.; Clark B. K.; Kim J.; Scuseria G. E. Multideterminant wave functions in quantum Monte Carlo. J. Chem. Theor. Comput. 2012, 8, 2181–2188. 10.1021/ct3003404. 26588949

[ref87] Giner E.; Scemama A.; Caffarel M. Fixed-node diffusion Monte Carlo potential energy curve of the fluorine molecule F2 using selected configuration interaction trial wavefunctions. J. Chem. Phys. 2015, 142, 044115 10.1063/1.4905528. 25637977

[ref88] Caffarel M.; Applencourt T.; Giner E.; Scemama A. Communication: Toward an improved control of the fixed-node error in quantum Monte Carlo: The case of the water molecule. J. Chem. Phys. 2016, 144, 151103 10.1063/1.4947093. 27389201

[ref89] Yao Y.; Giner E.; Li J.; Toulouse J.; Umrigar C. J. Almost exact energies for the Gaussian-2 set with the semistochastic heat-bath configuration interaction method. J. Chem. Phys. 2020, 153, 124117 10.1063/5.0018577. 33003731

[ref90] Scemama A.; Giner E.; Benali A.; Loos P.-F. Taming the fixed-node error in diffusion Monte Carlo via range separation. J. Chem. Phys. 2020, 153, 174107 10.1063/5.0026324. 33167659

[ref91] Benali A.; Gasperich K.; Jordan K. D.; Applencourt T.; Luo Y.; Bennett M. C.; Krogel J. T.; Shulenburger L.; Kent P. R.; Loos P.-F.; et al. Toward a systematic improvement of the fixed-node approximation in diffusion Monte Carlo for solids—A case study in diamond. J. Chem. Phys. 2020, 153, 184111 10.1063/5.0021036. 33187421

[ref92] Malone F. D.; Benali A.; Morales M. A.; Caffarel M.; Kent P. R. C.; Shulenburger L. Systematic comparison and cross-validation of fixed-node diffusion Monte Carlo and phaseless auxiliary-field quantum Monte Carlo in solids. Phys. Rev. B 2020, 102, 161104 10.1103/PhysRevB.102.161104.

[ref93] Carleo G.; Nomura Y.; Imada M. Constructing exact representations of quantum many-body systems with deep neural networks. Nat. Commun. 2018, 9, 5322 10.1038/s41467-018-07520-3. 30552316 PMC6294148

[ref94] Carleo G.; Troyer M. Solving the quantum many-body problem with artificial neural networks. Science 2017, 355, 602–606. 10.1126/science.aag2302. 28183973

[ref95] Nomura Y.; Darmawan A. S.; Yamaji Y.; Imada M. Restricted Boltzmann machine learning for solving strongly correlated quantum systems. Phys. Rev. B 2017, 96, 205152 10.1103/PhysRevB.96.205152.

[ref96] Nomura Y.; Imada M. Dirac-Type Nodal Spin Liquid Revealed by Refined Quantum Many-Body Solver Using Neural-Network Wave Function, Correlation Ratio, and Level Spectroscopy. Phys. Rev. X 2021, 11, 031034 10.1103/PhysRevX.11.031034.

[ref97] Viteritti L. L.; Rende R.; Becca F. Transformer Variational Wave Functions for Frustrated Quantum Spin Systems. Phys. Rev. Lett. 2023, 130, 236401 10.1103/PhysRevLett.130.236401. 37354409

[ref98] Casula M. Beyond the locality approximation in the standard diffusion Monte Carlo method. Phys. Rev. B: Condens. Matter Mater. Phys. 2006, 74, 161102 10.1103/PhysRevB.74.161102.

[ref99] Ten Haaf D. F. B.; Bemmel H. J. M. V.; Eeuwen J. M. J. V. I.; Saarloos W. V.; Ceperley D. M. Proof for an upper bound in fixed-node Monte Carlo for lattice fermions. Phys. Rev. 1995, 51, 13039 10.1103/PhysRevB.51.13039. 9978099

