# Supplementary material for: Beyond Single-Reference Fixed-Node Approximation in Ab Initio Diffusion Monte Carlo Using Antisymmetrized Geminal Power Applied to Systems with Hundreds of Electrons
Source: J Chem Theory Comput. 2024 May 24;20(11):4591–604. doi: 10.1021/acs.jctc.4c00139 (PMC11171267; doi:10.1021/acs.jctc.4c00139)
Supplement: Supplementary file 1 — ct4c00139_si_001.pdf [file ct4c00139_si_001.pdf]

Supplementary Material for Beyond single-reference fixed-node approximation in *ab initio* Diffusion Monte Carlo using antisymmetrized geminal power applied to systems with hundreds of electrons

Kousuke Nakano*
Center for Basic Research on Materials, National Institute for Materials Science (NIMS), Tsukuba, Ibaraki 305-0047, Japan and International School for Advanced Studies (SISSA), Via Bonomea 265, 34136, Trieste, Italy

Sandro Sorella
International School for Advanced Studies (SISSA), Via Bonomea 265, 34136, Trieste, Italy

Dario Alfè
Dipartimento di Fisica Ettore Pancini, Università di Napoli Federico II, Monte S. Angelo, I-80126 Napoli, Italy
Department of Earth Sciences, University College London, Gower Street, London WC1E 6BT, United Kingdom and Thomas Young Centre and London Centre for Nanotechnology, 17-19 Gordon Street, London WC1H 0AH, United Kingdom

Andrea Zen†
Dipartimento di Fisica Ettore Pancini, Università di Napoli Federico II, Monte S. Angelo, I-80126 Napoli, Italy and Department of Earth Sciences, University College London, Gower Street, London WC1E 6BT, United Kingdom
(Dated: May 21, 2024)

This file includes the supplementary information for the paper titled "Beyond single-reference fixed-node approximation in *ab initio* Diffusion Monte Carlo using antisymmetrized geminal power applied to systems with hundreds of electrons".

SUPPORTING RESULTS

Total energies and the number of variational parameters of hydrocarbons and fullerene

Table S-I shows the LRDMC total energies of eight hydrocarbons (CH_4, C_2H_4, C_2H_6, C_6H_6, $C_{10}H_8$, $C_{14}H_{10}$, $C_{18}H_{12}$, $C_{20}H_{10}$), and the C_{60} fullerene, obtained with different types of ansatz. Figure S-1 plots the LRDMC energy gains with respect to that obtained with the corresponding JSD ansatz. Table S-II shows the numbers of valence electrons and variational parameters for the 8 hydrocarbons and the C_{60} fullerene.

TABLE S-I. The total energies obtained by LRDMC with DLA ($a \to 0$) calculations for the 8 hydrocarbons (CH_4, C_2H_4, C_2H_6, C_6H_6, $C_{10}H_8$, $C_{14}H_{10}$, $C_{18}H_{12}$, $C_{20}H_{10}$) and the C_{60} fullerene. The units are in Hartree.

Ansatz	CH_4	C_2H_4	C_2H_6	C_6H_6	$C_{10}H_8$	$C_{14}H_{10}$	$C_{18}H_{12}$	$C_{20}H_{10}$	C_{60}
JSD, VMCopt [a]	-8.07878(3)	-13.71162(1)	-14.95337(6)	-37.61952(4)	-61.5103(3)	-85.4062(4)	-109.2998(4)	-119.3914(4)	-339.926(4)
JAGPn, FNopt [a]	-8.07945(3)	-13.71391(1)	-14.95438(6)	-37.62284(5)	-61.5150(3)	-85.4115(4)	-109.3059(6)	-119.3967(7)	-339.943(4)
JAGPn, VMCopt [a]	-8.07903(3)	-13.71319(1)	-14.95387(6)	-37.6224(1)	-61.5153(2)	-85.4116(4)	-109.3071(7)	-119.3976(7)	-339.937(5)
JAGP, VMCopt [a]	-8.07902(3)	-13.71408(4)	-14.95323(6)	-37.6202(1)	-	-	-	-	-
JSD, VMCopt [b]	-8.07869(7)	-13.71165(8)	-14.95331(5)	-37.6199(4)	-	-	-	-	-
JAGP, VMCopt [b]	-8.07949(7)	-13.71634(8)	-14.9544(2)	-37.6257(4)	-	-	-	-	-

[a] The small Jastrow basis sets ([$3s1p$] and [$3s$] for C and H atoms, respectively) are used.
[b] The large Jastrow basis sets ([$4s3p1d$] and [$3s1p$] for C and H atoms, respectively) are used.

Total energies of methane, water, and methane-water complexes

Table S-III shows the LRDMC total energies of methane, water, and methane-water complex. Hereafter, we discuss the role of molecular orbitals (MOs) or natural orbitals (NOs) in the AGPn ansatz. The comparison of the JSD energy

TABLE S-II. The numbers of valence electrons and variational parameters for the 8 hydrocarbons (CH_4, C_2H_4, C_2H_6, C_6H_6, $C_{10}H_8$, $C_{14}H_{10}$, $C_{18}H_{12}$, $C_{20}H_{10}$) and the C_{60} fullerene. The numbers are plotted in the Fig. 4 of the main body.

Formula	Num. valence electrons	Num. param. (JSD)	Num. param. (JAGPn)	Num. param. (JAGP)
CH_4	8	21	25	1651
C_2H_4	12	27	35	3729
C_2H_6	14	41	40	10841
C_6H_6	30	57	86	17629
$C_{10}H_8$	48	78	135	41568
$C_{14}H_{10}$	66	194	187	151066
$C_{18}H_{12}$	84	245	234	239987
$C_{20}H_{10}$	90	332	251	477318
C_{60}	240	422	647	1314634

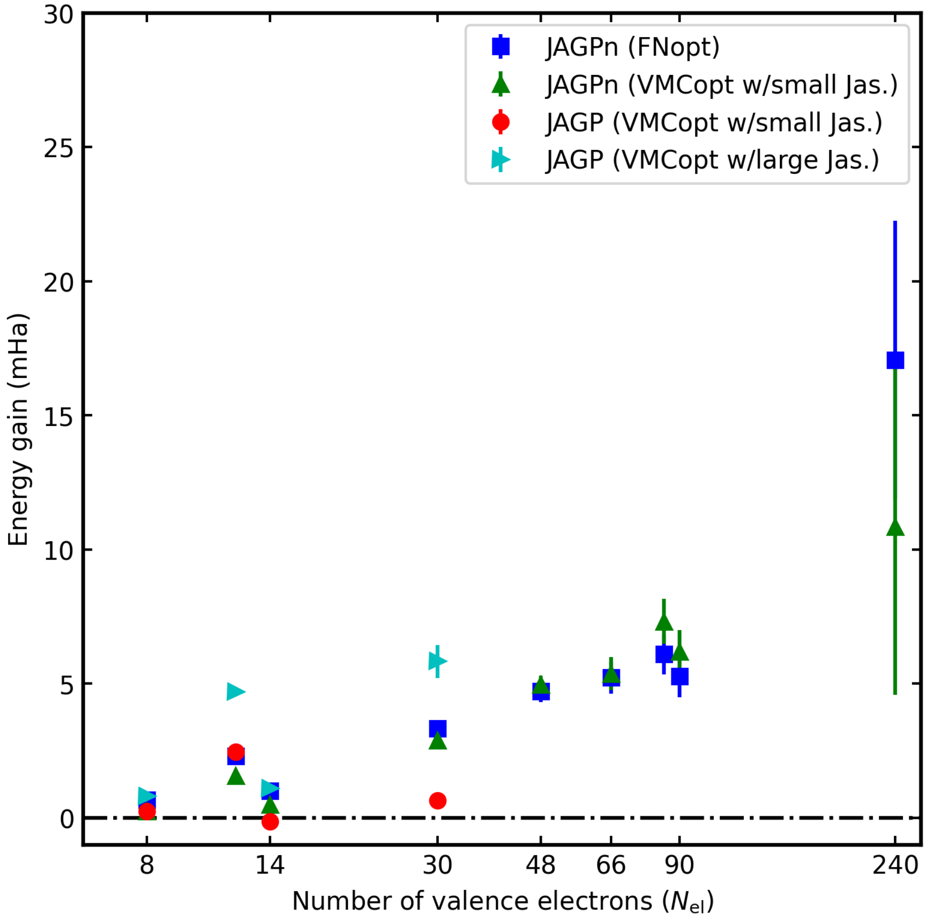

FIG. S-1. Improvements in the LRDMC energies ($a \to 0$), dubbed energy gain, of the JAGPn optimized with FN gradients (blue), JAGPn optimized with VMC gradients in the presence of the small Jastrow factor (green), JAGP optimized with VMC gradients in the presence of the small Jastrow factor (red), and JAGP optimized with VMC gradients in the presence of the large Jastrow factor (cyan) ansatze with respect to the traditional JSD ansatz for each of the considered systems, as a function of the number of valence electrons.

with the HF orbitals and that with LDA orbitals reveals that the nodal surface obtained by LDA is better than that obtained by HF. This is also true in the JAGPn ansatz. The comparison between JAGPn with the LDA(HF) orbitals and JAGPn with LDA(HF)-MP2 orbitals tells us the importance of MOs or NOs employed in the expansion of the JAGPn ansatz. Indeed, the total energies obtained with the JAGPn consisting of the LDA(HF)-MP2 orbitals are lower than those with the JAGPn consisting of the LDA(HF) orbitals for the three molecules. It indicates that the

NOs made from the MP2 calculations are better than the as-is LDA(HF) MOs in the expansion. This is because the MP2 virtual orbitals have more physical meanings than the LDA(HF) ones. The table also indicates that the NOs composed of the LDA orbitals are better than those composed of the HF orbitals. Thus, we concluded that the best strategy is making the JAGPn ansatz using the NOs composed of the LDA-MP2 orbitals, which are employed in the calculations reported in the main text. Table S-III also contains the results of the binding energy calculations for the methane–water dimer.

Table S-IV contains the total energies of the faraway water-methane complex, E_{faraway}, at a distance of ~ 11 Å and the sum of the isolated molecules, E_{isolated}, and the difference between them, $E_{\mathrm{SCE}} \equiv E_{\mathrm{isolated}} - E_{\mathrm{faraway}}$. They were computed with the JSD ansatz, with the JAGPn ansatz optimized using either VMC or FN gradients, and with the JAGP ansatz optimised with VMC gradients.

TABLE S-III. The total energies obtained by LRDMC ($a \to 0$) calculations for the methane, water, and the methane-water dimer.

Ansatz	MO	Opt.	CH_4 (Ha)	H_2O (Ha)	CH_4-H_2O (Ha)	Binding energy (meV)
JSD	HF	-	-8.07801(7)	-17.23413(8)	-25.31290(8)	-21(4)
JAGPn	HF	FNopt	-8.07821(7)	-17.23484(7)	-25.31373(7)	-19(3)
JAGPn	HF-MP2	VMCopt	-8.07820(8)	-17.23593(8)	-25.31605(7)	-52(4)
JAGPn	HF-MP2	FNopt	-8.07864(7)	-17.23630(8)	-25.31580(7)	-23(3)
JSD	LDA	-	-8.07858(3)	-17.23489(3)	-25.31445(4)	-27(2)
JAGPn	LDA	FNopt	-8.07909(3)	-17.23594(3)	-25.31600(4)	-27(2)
JAGPn	LDA-MP2	VMCopt	-8.07899(3)	-17.23693(3)	-25.31760(5)	-46(2)
JAGPn	LDA-MP2	FNopt	-8.07940(3)	-17.23718(3)	-25.31765(7)	-29(2)
JAGP	NA	VMCopt	-8.07902(3)	-17.23736(8)	-25.31789(8)	-41(3)

TABLE S-IV. Comparison of LRDMC energies (LRDMC, $a \to 0$) of the far-away water-methane complex and the sum of the isolated water and methane molecules, obtained with various ansatz. $E_{\mathrm{SCE}} = E_{\mathrm{isolated}} - E_{\mathrm{faraway}}$.

Ansatz	Opt.	E_{isolated} (Ha)	E_{faraway} (Ha)	E_{SCE} (meV)
JSD	-	-25.31347(4)	-25.31344(3)	-1(1)
JAGPn	VMCopt	-25.31592(4)	-25.31630(7)	10(2)
JAGPn	FNopt	-25.31658(4)	-25.31650(7)	-2(2)
JAGP	VMCopt	-25.31638(8)	-25.31679(7)	11(3)

Torsion energy of ethylene

Figure. S-2 shows the schematic figure of the ethylene torsion. The torsion energy is defined as the energy difference between the ground state ethylene (denoted as planer ethylene) and the orthogonally rotated ethylene (denoted as twisted ethylene). Table S-V shows the total energies of the ethylenes computed with the JSD and JAGPn ansatz. Table S-VI summarizes the obtained torsion energies and reference values obtained in previous works.

TABLE S-V. Comparison of LRDMC energies ($a \to 0$) of the planar and twisted ethylene.

molecule	JHF (Ha)	JAGPn-HF (Ha)	ΔE (mHa)
Planar Ethylene	-13.7099(5)	-13.7106(5)	-0.8(7)
Twisted Ethylene	-13.4977(5)	-13.5943(4)	-96.6(6)

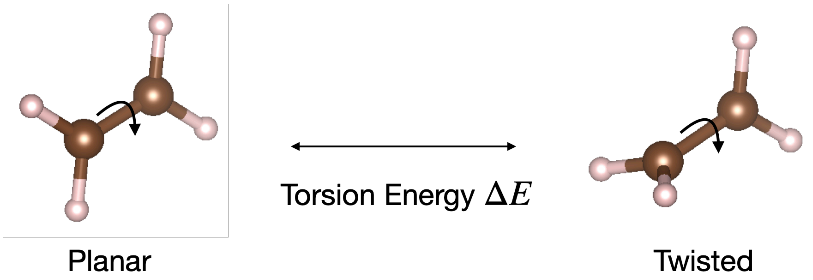

FIG. S-2. The schematic figure of the torsion between the planar and twisted ethylene.

TABLE S-VI. The torsion energies between the planar and twisted ethylene, obtained with various approaches.

Approach	ΔE (kcal/mol)
LRDMC/JHF	133.1(4)
LRDMC/JAGPn-HF	73.0(4)
LRDMC/JAGP[a]	70.2(2)
MR-CISD+Q[b]	69.2

[a] This value is taken from Ref. 1.
[b] This value is taken from Ref. 2.

* kousuke_1123@icloud.com
† andrea.zen@unina.it
[1] A. Zen, E. Coccia, Y. Luo, S. Sorella, and L. Guidoni, Journal of Chemical Theory and Computation **10**, 1048 (2014).
[2] M. Barbatti, J. Paier, and H. Lischka, The Journal of Chemical Physics **121**, 11614 (2004).
